# Co‐flowering with congeners does not affect buzz‐pollinator specialization and pollination performance in *Rhexia mariana*, but does affect floral trait variance

**DOI:** 10.1002/ajb2.70119

**Published:** 2025-10-28

**Authors:** Agnes S. Dellinger, Karolina Gwardiak, Ash Kerber, Viktoria C. Wieser, Karen D. Pérez‐Arroyo

**Affiliations:** ^1^ University of Vienna Rennweg 14 Vienna 1030 Austria; ^2^ Denver Botanic Gardens 1007 York Street Denver 80206 Colorado USA; ^3^ Escuela Nacional de Estudios Superiores Unidad Morelia Universidad Nacional Autonoma de México Morelia 58341 Michoacán México

**Keywords:** *Bombus impatiens*, buzz‐pollination, competition, facilitation, Melastomataceae, pollinator sharing, reproductive barriers

## Abstract

**Premise:**

Pollinator‐mediated plant‐plant interactions may be negative (i.e., competition, reproductive interference) or positive (i.e., facilitation). Especially when co‐flowering with close relatives (e.g., congeners), negative interactions through reproductive interference may be strong and result in floral trait divergence and increased pollination niche partitioning. However, when pollination services are limited, positive effects of pollinator sharing through floral trait similarity may outweigh the costs of reproductive interference. We therefore tested for evidence of negative or positive pollinator‐mediated plant‐plant interactions across a gradient of varying congeneric co‐flowering contexts in the genus *Rhexia* (Melastomataceae).

**Methods:**

We studied pollinator interactions, pollination performance and floral traits of *Rhexia mariana* across nine localities of varying cogeneric co‐flowering contexts (up to seven *Rhexia* species co‐flowering) in central Florida, USA.

**Results:**

Regardless of co‐flowering context, differential pollinator specialization was weak, with *Bombus impatiens* visiting all *Rhexia* species, removing pollen through buzz‐pollination. Co‐flowering context did not affect visitation rates or pollination performance, but the floral traits of *R. mariana* differed signficantly and were less variable in low compared to high co‐flowering contexts.

**Conclusions:**

We did not find support for either negative or positive effects of co‐flowering on pollination performance in *Rhexia mariana*, indicating that co‐flowering may instead have neutral effects. Negative effects of co‐flowering with close relatives hence do not seem to be strong enough to drive specialization on distinct buzzing bee pollinators in *Rhexia*. Sampling across more localities, paired with experimental approaches (e.g., manipulating co‐flowering density, assessing post‐zygotic reproductive barriers) will be essential to clarify whether reproductive interference through co‐flowering is indeed low.

Closely related (e.g., congeneric) plant species often overlap in several aspects of their ecological niche (Wiens, 2004), and may occur in the same habitat and be adapted to the same group of pollinators. When such co‐occurring congeners also overlap in phenology and so co‐flower, we may expect complex pollinator‐mediated plant‐plant interaction dynamics, with the potential for reproductive interference and reduced reproductive success (Baack et al., 2015; Tong and Huang, 2016; Ye et al., 2020; Johnson et al., 2022; Ivey et al., 2023; Pérez‐Barrales and Armbruster, 2023). Specifically, co‐flowering congeners may compete for pollinators, resulting in an overall reduction of pollinator visitation rates to each species (Figure 1). Furthermore, sharing pollinators may lead to increased rates of heterospecific pollen transfer (HPT), potentially resulting in stigma clogging or (infertile) hybrid seed formation (Moreira‐Hernández and Muchhala, 2019; Newman and Anderson, 2020). Such negative interactions may on the one hand lead to competitive exclusion of congeners from the community (Eisen and Geber, 2018; Johnson et al., 2022). Alternatively, negative interactions may lead to stronger partitioning of ecological resources through increased pre‐pollination reproductive barriers, such as through sub‐specialization on distinct pollinator species, individual pollinators, or pollinator body parts, or subtle temporal differentiation of anthesis (i.e., flowering at different times of day; Stone et al., 1996; Muchhala and Potts, 2007; Huang and Shi, 2013; Armbruster, 2014; Pérez‐Barrales et al., 2025). The intensity of such negative interactions can vary across the distribution range of a species, and pollination niche partitioning may be particularly pronounced in localities where multiple closely‐related species co‐flower (Figure 1; Armbruster and Muchhala, 2009).

**Figure 1:**
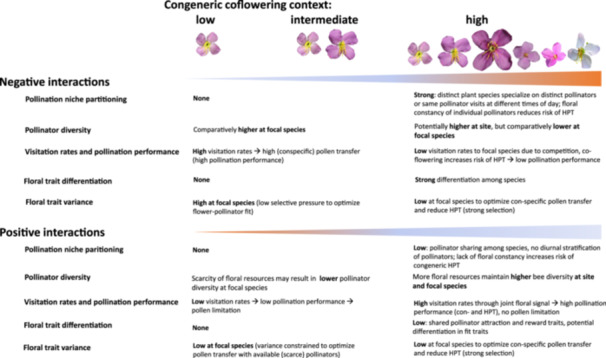
Conceptual framework of the potential negative (i.e., competition, reproductive interference) and positive (facilitation) pollinator‐mediated plant‐plant interactions across a gradient of congeneric co‐flowering intensity, with bar width indicating the strength of the interaction on the focal species—with orange indicating negative, blue indicating positive effects on focal species. Negative interactions among congeneric co‐flowering species: High co‐flowering intensity might lead to increased pollination niche partitioning (to reduce competitive interactions among plants) and reduced pollinator diversity at the focal species, leading to reduced pollinator visitation rates and lower pollination performance (reduced pollen transfer with additional heterospecific pollen transfer (HPT)); strong floral trait differentiation among species (to reduce competitive interactions) and low trait variance of the focal species (to optimize fit with pollinators) are expected. Low co‐flowering intensity, on the other hand, should not constrain pollination niches (potentially comparatively higher pollinator diversity at focal species) and high pollinator visitation rates may be expected, resulting in high rates of conspecific pollen transfer (absence of congeneric heterospecific pollen) and less constraints on floral traits, resulting in higher trait variability; floral trait differentiation may be absent or driven by other factors than co‐flowering congeners (i.e., adaptation to local polliantor pool [Pérez‐Barrales and Armbruster, 2023]). Positive interactions among congeneric co‐flowering species: High co‐flowering intensity may be characterized by high pollinator sharing (low niche partitioning) and potentially higher bee diversity (than at low co‐flowering intensity) and higher pollinator visitation rates, resulting in high pollination performance and low pollen limitation; floral traits involved in pollinator attraction such as color, display and reward are expected to be shared, but differentiation among traits mediating pollen placement and pickup (fit traits) may be expected; trait variance at the focal species may be low to optimize conspecific pollent ransfer. Under low co‐flowering intensities, pollinator abundance and diversity may be lower, resulting in lower visitation rates and pollination performance, and increased pollen limitation; floral traits may show low variance to optimize fit with the scarce pollinators.

Besides the negative effects of co‐flowering, co‐flowering with close relatives may also be beneficial if pollinator sharing increases reproductive success (Bergamo et al., 2020). Specifically, co‐flowering species may benefit from each other's presence because the overall increased floral display and resource availability may attract and sustain a larger and more diverse pool of pollinators (Figure 1; Ghazoul, 2006; Sargent and Ackerly, 2008; Bergamo et al., 2020; Wei et al., 2021). Such facilitative effects of co‐flowering are expected to be particularly important when pollinator services are scarce and plants would otherwise suffer from pollen limitation (Gurung et al., 2018; Gavini et al., 2021). In such instances, the benefits of receiving conspecific pollen alongside heterospecific pollen seem to outweigh the costs of heterospecific pollen receipt (Mesgaran et al., 2017; Wei et al., 2021). Particularly among closely related species, the receipt of such mixed pollen loads may select for strong post‐pollination reproductive barriers (i.e., interruption of heterospecific pollen tube growth, abortion of hybrid seeds, hybrid seed inviability, or hybrid male sterility) to minimize reproductive interference (Farnitano and Sweigart, 2023).

Floral trait differentiation to increase reproductive barriers and optimize conspecific pollen transfer may be key to allow co‐flowering with close relatives (Arceo‐Gómez and Ashman 2008; Eisen et al., 2020; Pérez‐Barrales et al., 2025). Competition for pollinators may lead to increased divergence in floral traits involved in pollinator attraction (i.e., color or scent) and traits involved in mediating fit with the pollinator (i.e., anther‐stigma distance; Newman et al., 2014; Opedal, 2019). While divergence in attraction traits might lead to specialization on different pollinators (and even shifts among functional pollinator groups, Fenster et al., 2004), fine‐tuning in traits mediating fit with the pollinator (also referred to as”fit traits”, determining where pollen is placed on and picked up from pollinators) might allow for pollen placement on different body parts even of the same, shared pollinator (i.e., for”differential use of the same pollinator”, Armbruster et al., 1994; Muchhala and Potts, 2007; Newman et al., 2014). Such trait differentiation has been demonstrated in various congeneric co‐flowering communities and should be particularly strong if receiving heterospecific pollen negatively impacts reproductive success in closely related species (Armbruster et al., 1994; Tong and Huang, 2016; Kooyers et al., 2017; Newman and Anderson, 2020; Figure 1). In contrast, if positive facilitation effects of co‐flowering outweigh negative impacts, we might expect convergence in attraction traits (i.e., joint pollinator attraction through shared color signals) even when traits mediating fit with pollinators diverge (Figure 1; Armbruster et al., 2004; Benitez‐Vieyra et al., 2007; Bergamo et al., 2020; de Jager et al., 2022).

Co‐flowering with close relatives may not only affect trait differentiation (trait means), but also trait variances, and, ultimately, plant reproductive performance (Dai et al., 2016). Specifically, strong negative effects may constrain co‐flowering relatives on narrow (distinct) phenotypic optima (optimal fit with distinct pollinators or pollinator body parts), reflected in low variances (low disparity) of traits mediating fit with pollinators (Armbruster et al., 2009; Pérez‐Barrales and Armbruster, 2023). Such low variances would become particularly apparent when comparing fit traits under variable co‐flowering scenarios, where fit traits are expected to be less constrained by selection and hence vary more under low than high co‐flowering intensity (Figure 1). When positive effects of co‐flowering prevail, low variance may be expected both in low and high co‐flowering intensity given the need to optimize fit with the fewer available pollinators under low co‐flowering, and avoidance of HPT under high co‐flowering intensity (Figure 1).

The genus *Rhexia*, which comprises 11 species in Melastomataceae, lends itself well to testing the impact of variable congeneric co‐flowering contexts on pollinator‐mediated plant‐plant interactions. Its center of diversity is in the southeastern USA (Appendix S1: Figure S1, S2), where up to eight species commonly co‐occur in the same habitat (Judd and Ionta, 2023). While most species show relatively narrow distribution ranges restricted to nutrient poor, sandy soils in Longleaf Pine savannas, some species, such as *Rhexia mariana* L., the focal species of this study, are widespread (Kral and Bostick, 1969). *Rhexia mariana* occurs in a wide range of habitats (i.e., moist roadsides, oak forests, wet meadows, pine savannas), and can be found in allopatry, as well as co‐flowering with up to six other *Rhexia* species (Figure 2A). All *Rhexia* species share the same, functionally specialized pollination strategy, buzz‐pollination by bees (Larson and Barrett, 1999). However, it remains unknown whether different *Rhexia* species specialize on distinct buzzing bees as a mechanism of reproductive isolation. Because pollen is released in large clouds during buzz pollination, scattering broadly on the bee's body, differential pollen placement on different body parts of the same bee seems unlikely as an isolating mechanism (compare Armbruster et al., 1994; Tong and Huang, 2016). Experimental crosses between *Rhexia* species have further shown incomplete post‐pollination reproductive barriers resulting in viable hybrid seeds, e.g., between *Rhexia mariana* and frequently co‐flowering *R. nashii* Small (Kral and Bostick, 1969; Judd and Ionta, 2023). Specialization on distinct buzzing bee species might thus be particularly important as a pre‐pollination reproductive barrier to reduce negative pollinator‐mediated plant‐plant interactions. On the other hand, an earlier study on *Rhexia virginica* L. (Larson and Barrett, 1999) in single‐species sites reported very strong pollen limitation, which, if also true for *R. mariana*, may render facilitative effects of co‐flowering and pollinator sharing particularly important.

**Figure 2:**
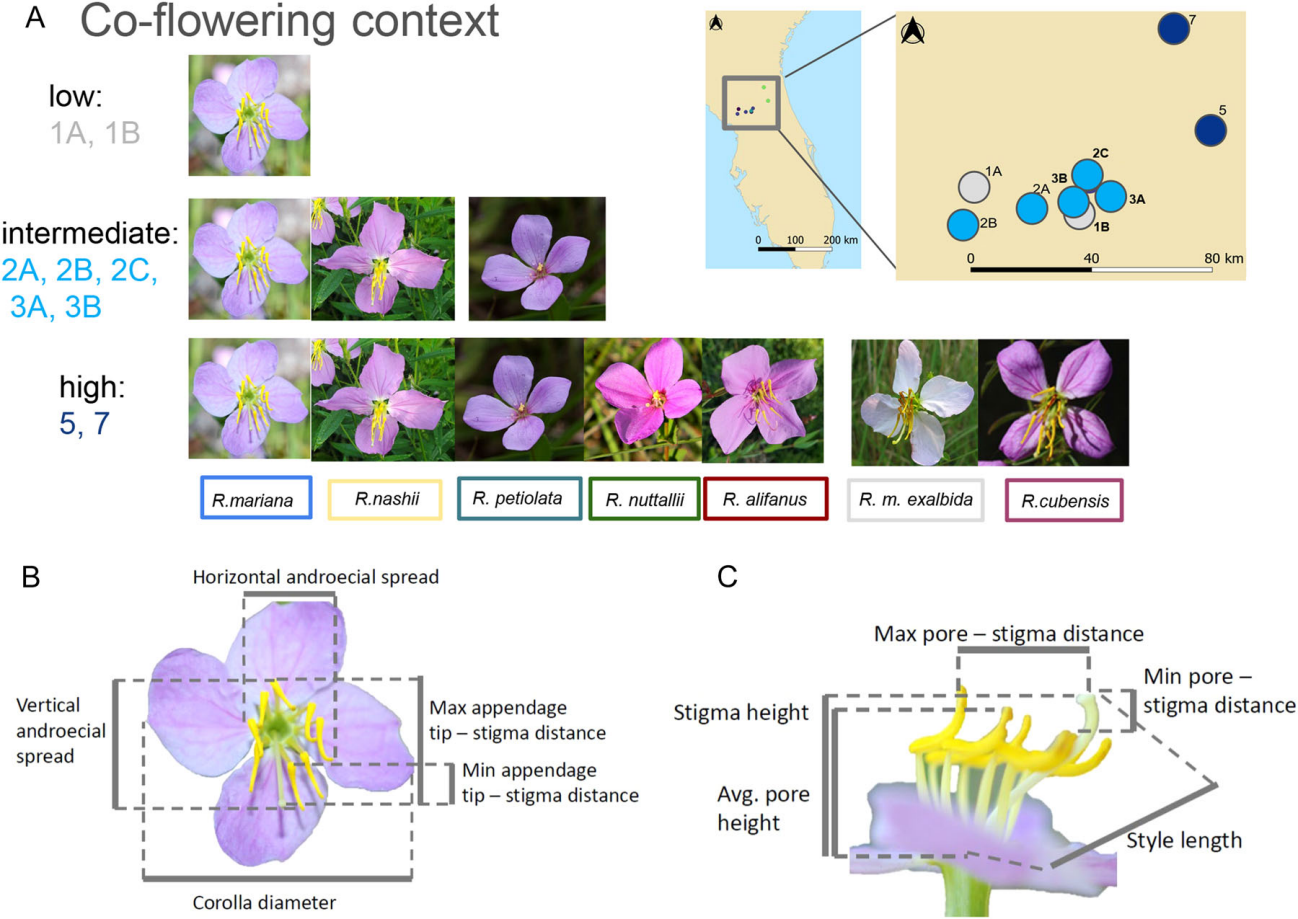
Sampling localities in Florida grouped by species composition and floral traits measured on *Rhexia mariana*. (A) *Rhexia mariana* occurrs in variable co‐flowering contexts, represented in our study by two localities of low co‐flowering context only featuring *R. mariana* (1A, 1B), intermediate co‐flowering context with *R. mariana* co‐flowering with large‐flowered *R. nashii* (2A, 2B, 2C) and with *R. nashii* and small‐flowered *R. petiolata* (3A, 3B), and high co‐flowering context, where *R. mariana* co‐flowered with *R. nashii, R. petiolata*, small‐flowered *R. nuttallii* and large‐flowered *R. alifanus* (5) as well as with large‐flowered *R. mariana var. exalbida* and *R. cubensis* (7). (B) Flower of *R. mariana* viewed from the front with five trait measures indicated, with corolla diameter and androecial spread potentially functioning as visual cues important in pollinator attraction, and maximum (max) and minimum (min) distances between appendage tips (where bees position their heads when buzzing) and stigmas describing size thresholds for potential pollinators (touching stigmas while buzzing). (C) Flower of *R. mariana* viewed from the side, with distance between pores and stigmas capturing where pollen is released in relation to the stigma.

In this study, we use *Rhexia mariana* as a model to investigate how co‐flowering with varying compositions of congeners (single‐species, two‐, three‐ and multi‐species sites, Figure 2A) affects differential pollinator specialization, visitation rates, pollination performance, and floral trait divergence. Focusing on closely related species (congeners that diverged between 1 mya for sister species, and 10 mya, the root age of *Rhexia*; Appendix S1: Figure S2) allows us to take a macroevolutionary perspective by comparing niche partitioning and specialization across *Rhexia*, while also evaluating how microevolutionary processes related to the direction of pollinator‐mediated interactions among *Rhexia* species (competition, facilitation, neutrality) affect *R. mariana* across varying co‐flowering contexts (Appendix S1: Table S1). Ultimately, this combination of a macroevolutionary perspective with a community‐level approach may help better understand how evolved differences among relatives play out in present‐day interactions, which may, in turn, have shaped the evolution of trait differentiation in the first place. Specifically, if co‐flowering increases negative pollinator‐mediated plant‐plant interactions (i.e., competition, reproductive interference), we expect increased floral trait divergence with reduced trait variance (as a mechanism to specialize on distinct bees), resulting in stronger pollination niche partitioning (specialization on distinct bees), reduced visitation rates and pollination performance at high co‐flowering intensity (Figure 1). However, if facilitative effects of co‐flowering prevail, we expect high pollination niche overlap, higher visitation rates and pollination performance, and high floral trait convergence (joint visual signalling) with low trait variance (to minimize reproductive interference) at multi‐species sites (Figure 1). We explore these hypotheses using a range of analyses. First, given that no pollinator observations were available for our study species, we had to establish whether all *Rhexia* species share pollinators. We additionally quantified how often pollinators transitioned among *Rhexia* species at one multi‐species site to assess potential for reproductive interference. Second, to test how pollination performance varies with co‐flowering context, we quantified visitation rates, pollen removal from stamens and pollen deposition on stigmas in *Rhexia mariana* across all nine study localities. Third, to explore the effects of co‐flowering on floral traits, we compared ten pollination‐relevant floral traits across all species and additionally calculated variance in floral traits of *R. mariana*.

## MATERIALS AND METHODS

### Study sites and plant species

We conducted fieldwork in nine localities around Gainesville, Florida, USA (Figure 2A; Appendix S1: Table S2). Species richness of the genus *Rhexia* is highest in the southeastern US, with up to eight species co‐occurring in the same habitat (Appendix S1: Figure S1). Our study localities differ in species composition from sites only featuring our main study species *Rhexia mariana* (sites 1A, 1B, classified as low co‐flowering intensity), to sites containing *R. mariana* and *R. nashii* (sites 2A, 2B, 2C, classified as intermediate co‐flowering intensity), to sites containing *R. mariana, R. nashii*, and *R. petiolata* Walter (sites 3A, 3B, classified as intermediate co‐flowering intensity), and to our most species‐rich sites 5 (*R. mariana*, *R. nashii*, *R. petiolata*, *R. nuttallii* C.W.James, and *R. alifanus* Walter) and 7 (*R. mariana*, *R. nashii*, *R. petiolata*, *R. nuttallii*, *R. alifanus*, *R. cubensis* Griseb., and *R. mariana var. exalbida* Michx.), also classified as high co‐flowering intensity. We also found *Rhexia lutea* Walter in site 9, but this species flowers earlier in the season, and so was not included in our assessments. All localities had sandy soils, but the vegetation composition differed among study sites. The most species‐rich sites 5 and 7 were in pristine longleaf pine savannas, sites 1A and 2C featured a mix of pine flatwoods and oak forests, while sites 1B, 2A, 2B, 3A and 3B represented relatively open patches of meadow (along water bodies or roadsides) with adjacent pine and oak forests. Note that distances among sites varied between 2.7 km and 105 km (Appendix S1: Table S3), with the shortest distances falling within the foraging ranges of bumblebees (mostly below 1.5 km, but occasionally larger ranges reported for European species; Knight et al., 2005). To assess potential bias through geographic clustering, we ran Mantel tests, which allow for testing of correlations between two pairwise distance matrices (see Visitation rates and pollination performance).

All co‐flowering *Rhexia* species studied here have pink corollas with contrasting yellow stamens (Figure 2). However, there are overall differences in petal and stamen size, and in stamen arrangement. Specifically, *R. mariana* belongs to a group (including *R. alifanus, R. nashii, R. cubensis*, and *R. mariana var. exalbida*) of relatively large‐flowered species with long, flexed stamens (Figure 2B). The two remaining co‐flowering *Rhexia* species (*R. petiolata, R. nuttallii*) are small‐flowered with short, straight stamens (Figure 2C). Note that these morphological differences do not seem to depend on phylogenetic relatedness (Appendix S1: Figure S2; Penneys et al., 2022; Reginato et al., 2022), in that small‐flowered *R. petiolata* may be sister to all other species (but also see treatments by Ionta et al., 2007), and large‐flowered *R. alifanus* may be sister to small‐flowered *R. nuttallii*. *Rhexia mariana* seems to be sister to *R. cubensis*, while *R. nashii* belongs to a separate clade (Appendix S1: Figure S2). The crown age of *Rhexia* is estimated to be around 10 mya, and crown ages of sister species are only about 1 mya. Our comparison of “congeneric” species thus includes a range of ages, with the crown age of *R. mariana‐R. cubensis* being only about 1 mya, while a common ancestor of *R. mariana* and *R. petiolata* would have the crown age of the genus (Appendix S1: Figure S2). *Rhexia mariana* has been reported to be self‐incompatible but can also reproduce through apomixis (Kral and Bostick, 1969), which we confirmed through preliminary crossing experiments (Dellinger, unpublished data).

### Pollination niche partitioning: Pollinator composition, diurnal stratification, constancy, and pollinator diversity

We conducted fieldwork in all our study sites in July 2022 in teams of two to three researchers (Appendix S1: Table S2). We visited sites 1B, 2A, 2C and 3B once, sites 1A, 2B, 3A, and 5 twice, and the most species‐rich site 7 three times. We made repeated visits if we could not obtain pollinator observations or trait measurements for all species within a single day (e.g., because of many co‐flowering species, or unexpected rain or heat episodes ending pollinator visits). We only included pollinator data from days with good, dry weather. To account for differences in sampling intensity among sites and species, we scaled the number of pollinators by the number of flowers observed. We arrived at each fieldsite at dawn before sunrise (at around 5:45 AM), which is before bee visitation began at around 6:00 AM.

To explore whether co‐flowering sites have higher pollination niche partitioning, indicative of negative (competitive) interactions, we performed empirical pollinator observations on all *Rhexia* species at each site. We collected the pollinator data presented here using a combination of three different techniques. First, we performed direct observations of pollinators by selecting different patches of *Rhexia* species at each site. At each patch, we observed pollinators for 30 minutes, noting the number of flowers per species, and each insect visiting the flowers. For these initial assessments, we grouped the visiting insects into morphospecies that we could reliably determine in the field (i.e., *Bombus pensylvanicus* De Geer, *Bombus* sp. 2, halictid bee, megachilid bee, tiny bee). We noted the behavior of the respective visiting insect (buzzing vs. not buzzing) and whether or not it touched the stigma while buzzing the stamens (and hence could act as pollinator). Buzzing behavior is easily determined by the buzzing sound emitted by the bee, and its typical crouching position over the stamens. We switched to a different patch after 30 minutes and repeated these observations until visitation rates died off during the midday heat at around 11:00 AM.

Second, to expand total observation hours across all *Rhexia* species, we employed additional video monitoring. We placed Sony CX450 camcorders (Sony Group, Tokyo, Japan) on tripods in front of a *Rhexia* individual at ~1 m distance to not disturb pollinators and filmed the flowers for the entire field day (i.e., from around 6:00 AM to 11:00 AM). We used the Sony software PlayMemoriesHome (https://support.d-imaging.sony.co.jp/support/app/playmemorieshome/de/index.html) to review the videos at 2× speed, noting how many flowers were filmed, and each flower visit. We recorded insect morphospecies and behavior the same way as in direct observations.

Third, to identify pollinators more accurately, we performed transect walks at each study locality between 6:00 AM and 11:00 AM, taking care to perform transect walks in different areas than direct observations to avoid biasing results. During each walk, we slowly walked for around 10 minutes within each locality, noting the number of flowers of all *Rhexia* species growing within 1 m to each side of the walking person. If we encountered a floral visitor on one of these flowers, we noted down the insect morphospecies and captured it with an entomological net and transferred it into a live‐viewing catching jar. For bumblebees, we measured body length for 116 individuals using digital calipers, immobilizing bees in the catching jars by squeezing them gently with the foam lid. Finally, we selected a subset of the individuals caught per morphospecies for detailed identification, euthanized the respective individuals in killing jars, and released all other individuals. We preserved all euthanized bees by freezing them at –20°C until mounting them. We identified bees to species or genus level with the help of bee expert Dr. Adrian Carper (University of Colorado, Boulder, Colorado, USA). We pooled data from direct and video observations with data from transect walks to determine pollinator composition (i.e., for buzz pollination, all insects buzzing flowers and touching the stigma) for each *Rhexia* species at each locality.

Although flower visitation of multiple plant species by the same pollinator species is generally thought to be equivalent to pollinator sharing, recent studies have shown that individuals of the same pollinator species can be highly specialized (Carneiro et al., 2024). Such individual‐level specialization may significantly reduce chances of heterospecific pollen flow, and thus reproductive interference. Therefore, to explore whether specialization occurs at the level of individual bees in *Rhexia*, we quantified floral constancy of the most frequent buzz‐pollinating bee on *Rhexia mariana*, *Bombus impatiens* Cresson, in locality 7 on four different days in July 2023. We selected five patches of around 10 × 2 m along a sandy trail containing multiple co‐flowering *Rhexia* species and counted the number of open flowers of each species on each day. When an individual of *Bombus impatiens* arrived at a patch, we followed it throughout the patch and noted down the sequence of flowers visited. We recorded foraging paths of 133 *Bombus impatiens* individuals across a total of 335 flowers (including 112 flowers of *R. mariana*). Note that we chose locality 7 because it hosted the most *Rhexia* species and so was most informative for exploring the probability of interspecific congeneric pollen flow (with potential negative impacts through reproductive interference). While we did not quantify constancy at the other co‐flowering localities, we have also seen regular interspecific moves by *B. impatiens* at the other localities.

To estimate pollinator constancy at locality 7, we summed up all the inter‐ and intraspecific transitions of each individual, and calculated flower constancy following Gegear and Thomson (2024) using the constancy index, *CI* (Jacobs, 1974), calculated as *CI* = *(c‐e)/(c* + *e‐2ce)*, where *c* is the proportion of intraspecific moves (within *R. mariana*) of an individual bumblebee and *e* is the proportion of intraspecific moves (within *R. mariana*) expected based on the overall visitation frequency of *R. mariana*. Specifically, if *p* is the proportion of visits to *R. mariana*, then *e* = *p²* + *(1‐p)²*. The constancy index ranges between −1 (no constancy) to 0 (random moves) to 1 (full constancy). To visualize transition patterns between *R. mariana* and all other species, we calculated a transition rate matrix by dividing the number of all observed transitions by the relative abundance of flowers of each species in the selected patch. We used heatmaps to visualize the transition rate matrix among all species.

To evaluate whether pollinator diversity differs among co‐flowering contexts, we calculated Shannon diversity both at the site level (across all *Rhexia* species) and for *R. mariana* only using the function diversity in the R package vegan (Oksanen et al., 2022). We further compared evenness by dividing Shannon diversity by the log of species numbers per grouping variable.

### Visitation rates and pollination performance

To explore whether co‐flowering sites have higher visitation rates, potentially indicative of facilitative effects, we calculated visitation rates per flower per hour for each *Rhexia* species at each locality. We pooled both the data from direct and video observations (Appendix S1: Table S4). We tested whether co‐flowering context affects visitation rates to *R. mariana* using Generalized linear mixed models (GLMMs), with visitation rate as dependent variable, co‐flowering context as a predictor, and site as random effect grouping variable. Given the zero inflation in our data caused by low visitation rates, we fit Hurdle models with a Gamma distribution for the conditional model and separately accounting for zero‐inflation using the R package glmmTMB (Brooks et al., 2017). Further, for co‐flowering sites, we tested whether visitation rates differed among *Rhexia* species, again specifying site as random effect grouping variable.

To assess whether co‐flowering context affects pollination performance of *R. mariana*, we compared male (pollen remaining in stamens) and female (stigmatic pollen loads) pollination performance (Opedal et al., 2023) across localities. Note that by using pollination performance (sensu Opedal et al., 2023), we explicitly focus on the pollen export and pollen receipt pathway, but not reproductive fitness. This is important because pollen grains of all *Rhexia* species look alike, and it is impossible to quantify HPT from stigmatic pollen loads. Therefore, our assessments only quantify absolute pollen receipt on stigmas. Because *Rhexia marina* has been reported as self‐incompatible (Kral and Bostick, 1969), and autonomous self‐pollen deposition is not possible (stamens require vibrations to shed pollen), stigmatic pollen loads reflect pollen deposition by floral visitors.

When pollinator visitation ceased at around 11:00 AM, we collected all eight stamens and the style from approximately 15 randomly selected flowers of *R. mariana* per study site into separate 1.5 ml Eppendorf tubes filled with 70% ethanol. To assess male pollination performance, we counted the number of pollen grains left in single stamens following Dellinger et al. (2019). We randomly selected three stamens per flower and separated them into 1.5 ml Eppendorf tubes filled with 1 ml of purified water. To remove the remaining pollen from the anthers, we gently mascerated the stamens using a pestle. To ensure complete removal of all pollen, we then placed the macerated samples into an ultrasound bath (similar frequencies to bee vibrations) for 15 minutes. Because pollen may settle at the bottom of the tube, we resuspended pollen by gently pipetting the liquid in the tube before counting. Next, we injected 100 µl of sample solution into a liquid particle counter (Topas FAS B, Topas GmbH, Dresden, Germany), which separates particles into 62 size classes (2–200 µm). To establish which size classes contained the pollen grains from our samples, we first measured pollen grains of *R. mariana* under a light microscope (mean 20.25 µm, min 14 µm, max 25 µm). Correspondingly, we selected 7 size classes covering particle sizes from 13.9 to 21.8 µm. Note that these size classes are slightly smaller than the maximum pollen grain diameter measured under the microscope. Because *Rhexia* pollen grains are tricolpate and not round, they may get hit by the laser beam in the particle counter at different angles and therefore appear smaller. Finally, we multiplied the pollen counts by ten to arrive at total stamen pollen counts, and averaged counts across the three stamens per flower.

To estimate stigmatic pollen loads, we carefully sliced the stigma off the style and put it into a drop of 10% lactic acid to soften the tissue. After one minute, we placed a coverslip on top of the stigma and gently flattened out the stigma. We placed the sample under a light microscope with a filter for fluorescent light and measured the total flattened‐out stigmatic area at 4x magnification. Next, we changed to 20× magnification and selected three patches of the stigmatic surface at random (making sure to capture both highly sparsely covered areas if stigmas presented high variability), measured the area of the stigmatic surface in these patches and counted all pollen grains. Finally, we calculated how many pollen grains were found on average per µm² and multiplied this number by the total stigmatic area measured at 4x magnification (see Dellinger et al., 2019 for details). Pollen grains of all *Rhexia* species are small (~20 µm in diameter), tricolpate, and with a mostly smooth surface. Our estimates of pollen deposition on *R. mariana* can therefore only represent overall differences in pollination performance, but do not capture relative proportions of con‐ versus heterospecific *Rhexia* pollen transfer. We did, however, exclude non‐*Rhexia* pollen that looked substantially different (i.e., larger, ornamented) from these pollen counts.

To test whether co‐flowering context affects pollination performance, we fit GLMMs with a Gamma distribution to accomodate the right‐skewed data, treating pollen remaining in stamens and pollen deposited on stigmas as response variables, co‐flowering context as a predictor and study site as a random effect grouping variable using the glmmTMB function (Brooks et al., 2017). We further tested whether locality‐level pollination performance could be explained by visitation rates using a generalized linear model (GLM) with a Gamma distribution. Finally, to assess potential impacts of spatial clustering of study sites, we used Mantel tests on pairwise site differences for visitation rates, pollination performance, and geographic distance.

### Floral traits across *Rhexia* species and co‐flowering contexts

To test whether multi‐species sites have higher floral trait divergence (indicative of competitive effects) or lower trait divergence (indicative of facilitative effects) among co‐flowering *Rhexia* species, we measured ten floral traits of functional relevance during the pollination process from all species occurring at each site (Figure 1B, C). Two traits (corolla diameter and spread of the visually contrasting stamens) are important in pollinator attraction, while the remaining eight traits (i.e., distances between stigma and stamen appendages which bees grasp when they apply buzzes, distances to the stamen pores which release pollen) are critical in mediating fit with the pollinators (Dellinger et al., 2023). We used digital calipers to measure these traits on 15 flowers per species per study site. We used principal component analyses (PCAs, with prcomp function in base R) to visualize differences in flower morphology among species. We used permutational analysis of variance (PERMANOVAs, with 999 permutations) to test for significant differences in flower morphology among species across all sites, and among species within multi‐species sites (*adonis2* function, *vegan*; Oksanen et al., 2022). For sites with more than two species, we used the function *pairwiseAdonis* (Martinez Arbizu, 2017) as post‐hoc analysis to determine which species differed significantly from each other, employing Bonferroni correction to account for multiple comparisons.

To assess whether co‐flowering context affects floral trait divergence of *Rhexia mariana*, we ran a PERMANOVA on measurements of *R. mariana* only and visualized floral trait space occupation using PCA. We further quantified variance (disparity) of flowers of *R. mariana* across co‐flowering contexts to test whether co‐flowering is associated with reduced floral trait variances (potentially indicative of selection to reduce negative plant‐plant interactions). We used the function *betadisper* (*vegan*), calculating the mean distance to each context's centroid, and Tukey's HSD as post‐hoc test. Because we have more localities in the “intermediate” co‐flowering category (five) than in either “low” or “high” (two each), we also ran disparity tests comparing across the different localities. To establish whether trait variances of *R. mariana* differ from community‐level trait variances, we calculated community‐weighted dispersion for the whole dataset, co‐flowering localities only and co‐flowering localities but removing *R. mariana* using function cwd in R‐package BAT (Cardoso et al., 2025). Finally, we ran Mantel tests to test for significant associations in trait means and SD across space.

## RESULTS

### Pollination niche partitioning: Pollinator composition, diurnal stratification, and constancy

**Figure 3:**
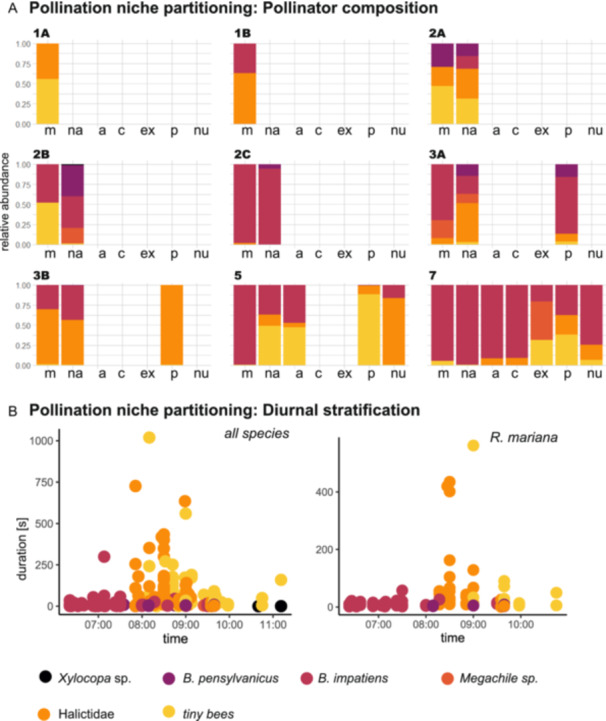
Pollination niche partitioning was low across sites and species (A), with the most important large bumblebee pollinators visiting flowers of all species before small bees visited (B). **(**A) Co‐flowering intensity (indicated by different colors for 1A, 1B – low, 2A – 3B – intermediate, 5, 7 – high) did not affect differential pollinator specialization and pollinator diversity, with a site with intermediate co‐flowering intensity (2B) having the highest pollinator diversity (on *R. nashii*). The large bumblebees *Bombus impatiens* and *B. pensylvanicus* can be considered legitimate and important pollinators across all species because they commonly touch the stigma while buzzing stamens. (B) Diurnal stratification of bee visits to all species and only to *R. mariana*, pooled across all study sites. Large bumblebees start visiting flowers right at sunrise when ambient temperatures are still low and spend only a few seconds buzzing each flower, then moving swiftly to another flower. Smaller bees (Halictidae and very small black bees (e.g., subgenus *Dialictus*)) may be considered secondary pollinators or pollen robbers (often buzzing single stamens and rarely touching the stigma) in large‐flowered *Rhexia* species, while they also act as primary pollinators and show higher relative abundances in small‐flowered species (*R. nuttallii*, *R. petiolata*) with shorter stamens and smaller distances between stamen pores and stigmas. These smaller bees start visiting flowers later in the day (when ambient temperatures are higher) and spend more time (often more than a minute) on each flower buzzing single stamens. m – *R. mariana*, na – *R. nashii*, a – *R. alifanus*, c – *R. cubensis*, ex – *R. mariana var. exalbida*, p – *R. petiolata*, nu – *R. nuttallii*.

To establish whether the different *Rhexia* species are specialized to pollination by different bees, which we expect to be particularly important under high co‐flowering intensitiies if negative pollinator‐mediated plant‐plant interactions are common, we documented pollinator composition across the nine study sites. Against expectations, we found little variation in pollinator composition across localities, and no relation to co‐flowering context (Figure 3). Bumblebees were the most important primary pollinators of *Rhexia mariana*, with *Bombus impatiens* being the most frequent pollinator, and *B. pensylvanicus* visiting *R. mariana* in two localities (1B, 2 A; Figure 3). *Bombus impatiens* also visited all other *Rhexia* species, and *B. pensylvanicus* also visited large‐flowered *R. nashii* and small‐flowered *R. petiolata*. We observed megachilid bees (*Megachile mendica* Cresson) in three localities (2B, 3 A, 7) on *Rhexia mariana*, *R. nashii*, *and R. mariana* var. *exalbida*, while we saw carpenter bees (*Xylocopa* sp.) only in one locality (2B) on *R. nashii*. These large bees can be considered primary pollinators in all *Rhexia* species because they touch the stigma while grasping the stamens with their legs and mandibles when applying buzzes. We further observed smaller bees of the family Halictidae (*Lasioglossum* sp., *Augochlora aurata* Smith, *A. pura* Say, and *Agapostemon splendens* Lepeletier) visiting all *Rhexia* species (Figure 3). In the larger‐flowered *Rhexia* species, these smaller bees should be considered secondary pollinators only because they generally do not touch stigmas while buzzing. The smallest bees (e.g., *Lasioglossum* subgen. *Dialictus*) often only buzz the tips of stamens and might hence be considered pollen robbers. In the smaller‐flowered species *R. petiolata* and *R. nuttallii*, however, where stamens and styles are much closer together, these small bees might also be considered important primary pollinators.

**Figure 4:**
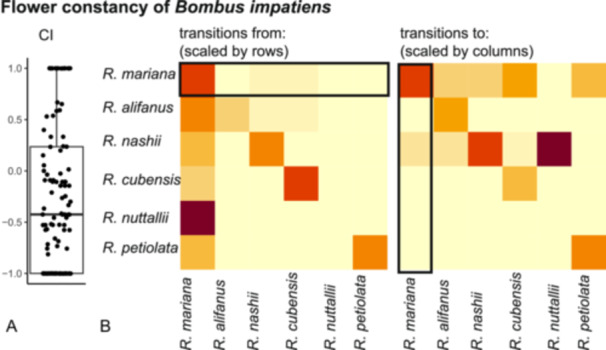
Low floral constancy of *Bombus impatiens* at high‐intensity co‐flowering locality 7 indicates that *Rhexia mariana* may receive substantial amounts of heterospecific pollen when co‐flowering. **(**A) Floral constancy estimated from 109 individuals of *B. impatiens* at locality 7 was relatively low (median CI −0.4), with 85% of individuals performing at least some interspecific moves. (B) The transition rate matrix shows that a high proportion of flower transitions are conspecific (i.e., from *R. mariana* to *R. mariana*), but also that transitions from other co‐flowering species to *R. mariana* occur. Proportion of transitions from *Rhexia* species (first panel, rows have been scaled by each species’ sum of transitions) and proportion of transitions to *Rhexia* species (second panel, columns have been scaled by each species’ sum of transitions), all transitions are scaled by floral abundance of each *Rhexia* species. Red colors indicate a high proportion of transitions, yellow colors indicate a low proportion of transitions.

Next, we tested whether the different *Rhexia* species are visited at different times of day, with a diurnal stratification of the pollinator community potentially aiding in subtle pollinator specialization (especially important if negative interactions are common at high‐intensity co‐flowering sites). We did indeed observe a diurnal stratification in the pollinator community, with large bees (e.g., *Bombus*) visiting flowers earlier (from 6:00 AM onwards) than small bees (e.g., Halicitidae, from 8:00 AM onwards, Figure 4A, B). This diurnal stratification was unrelated to co‐flowering context, however, and is found for all *Rhexia* species and sites (Appendix S1, Figure S4). The large bees also moved more quickly between flowers and spent less time per visit (median visit duration: *Xylocopa* 1 s, *B. pensylvanicus* 5.5 s, *B. impatiens* 6 s, *Megachile* sp. 6 s) than the smaller bees (median visit duration: Halictidae 33 s, tiny bees 55 s), likely rendering them particularly important pollinators.

Given that *Bomus impatiens* was the shared main pollinator across *Rhexia* species and sites, we also quantified whether individual *B. impatiens* bees show floral constancy at high‐intensity co‐flowering locality 7. A lack of constancy would be indicative of high potential for congeneric heterospecific pollen transfer, substantially increasing the potential for reproductive interference. *Bombus impatiens* showed relatively low floral constancy (CI –0.42 ± 0.7) on *Rhexia mariana* in locality 7 (Figure 4C). Of a total of 109 bumblebee individuals, 30 (28%) showed complete inconstancy, making interspecific moves at each new flower visit, while 16 (15%) showed complete constancy (only making intraspecific moves). Across all *Rhexia* species, *R. mariana* had the highest proportion (72%) of intraspecific moves (scaled by flower abundance), followed by large‐flowered *R. cubensis* (69%) and *R. nashii* (51%) and small‐flowered *R. petiolata* (50%). Large‐flowered *R. alifanus* only received 29% of intraspecific moves, and we did not observe any intraspecific moves of *Bombus impatiens* in small‐flowered *R. nuttallii* (Figure 4D). The highest proportion of interspecific moves from *R. mariana* occurred to similarly large‐flowered *R. nashii* (13%) and *R. cubensis* (11%), while only about 2% occurred to each large‐flowered *R. alifanus* and small‐flowered *R. petiolata. Rhexia mariana* received the highest proportion of interspecific moves from large‐flowered *R. nashii* (21%) followed by *R. alifanus* (6%), *R. cubensis* (3%) and small‐flowered *R. petiolata* (2%) and *R. nuttallii* (0.5%). While not quantified, we regularly observed interspecific moves also at the other co‐flowering study localities, indicating that this pattern of partial inconstancy is not unique to locality 7.

### Pollinator diversity

We found higher Shannon diveristy on *R. mariana* in low (0.75) than high (0.11) co‐flowering intensity, which could be indicative of negative interactions (competition) reducing pollinator diversity (Figure 1). Accordingly, at high co‐flowering intesity, site‐level Shannon diversity was higher (0.68) than Shannon diversity on *R. mariana* (Appendix S1: Figure S3, Table S5). These differences in Shannon diversity are mostly due to the dominance of *B. impatiens* on *R. mariana* in high co‐flowering intensity (reflected in low evenness 0.09), while smaller (halictid) bees were more common in low co‐flowering intensity (reflected in higher evenness: low – 0.54, Figure 3). Generally, intermediate co‐flowering intensity showed highest Shannon diversity (site‐level 1.26, *R. mariana* 1.25) and evenness (0.78).

### Visitation rates and pollination performance

**Figure 5:**
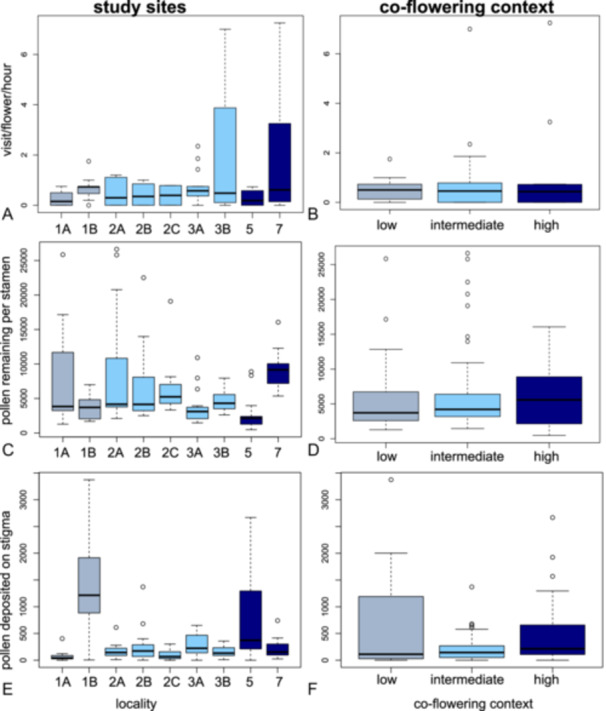
Visitation rates and pollination performance of *Rhexia mariana* across sites and co‐flowering contexts. (A, B) Visitation rates to *Rhexia mariana* were low overall and did neither show a reduction with increased co‐flowering intensity (indicative of plant‐plant competition for pollinators) nor an increase (indicative of facilitative effects through joint signalling). (C, D) Pollination performance as measured through pollen remaining in stamens did not differ across co‐flowering contexts, pollen removal was independent of visitation rates. (E, F) Pollination performance as measured through pollen deposited on stigmas did not show any relationship with co‐flowering context and appeared mostly unrelated to visitation rates.

Testing whether sites with high co‐flowering intensity show lower visitation rates to *R. mariana* due to competition, or higher visitation rates due to facilitation than single‐flowering localities (Figure 1), we found no effect of co‐flowering context on visitation rates (Figure 5A; conditional model, i.e., low‐high co‐flowering context z‐value = 1.686, p = 0.09, intermediate‐high co‐flowering context z‐value 1.067, p = 0.26, zero‐inflation model, i.e., low‐high co‐flowering context z‐value = –1.245, p = 0.22, intermediate‐high co‐flowering context z‐value = –0.534, p = 0.59). We recorded the lowest median visitation rates (0.15 and 0.19 visits/flower/hour) on both a single‐ and a multi‐species site (1A, 5), while we found the highest visitation rates (0.73, 0.61) on a single‐ and a multi‐species site (1B, 7). Visitation rates did not differ among *Rhexia* species (Appendix S1: Table S4).

To test whether co‐flowering decreases (i.e., due to competition) or increases (i.e., due to facilitation) pollen transfer, we compared the amount of pollen remaining in stamens (male pollination performance) and pollen deposited on stigmas (female pollination performance). As with visitation rates, co‐flowering context did not affect pollination performance in *Rhexia mariana*, but study localities differed significantly from each other (Figure 5B, C; Appendix S1: Table S6, S7, S8). The two sites with the highest *Rhexia* diversity had both the lowest and highest median amount of pollen remaining (locality 5: 2143 (±3080) pollen grains vs. locality 7: 9251 (±2962) pollen grains), while the two single‐flowering localities had both the lowest and highest median number of pollen grains deposited on stigmas (locality 1A: 40 pollen grains, locality 1B: 1326 pollen grains). Visitation rates by bees did not explain male (estimate = –2.3e‐14, t‐value = –1.63, p = 0.14) or female pollination performance (estimate = –0.013, t‐value = –2.28, p = 0.06; Appendix S1: Figure S5). Similarly, distances among populations did not correlate significantly with pairwise differences in visitation rates (R² = 0.1, p = 0.3) or stigmatic pollen loads (R² = –0.14, p = 0.64), but with pollen remaining (R² = 0.7, p = 0.02).

### Floral differentiation among *Rhexia* species

**Figure 6:**
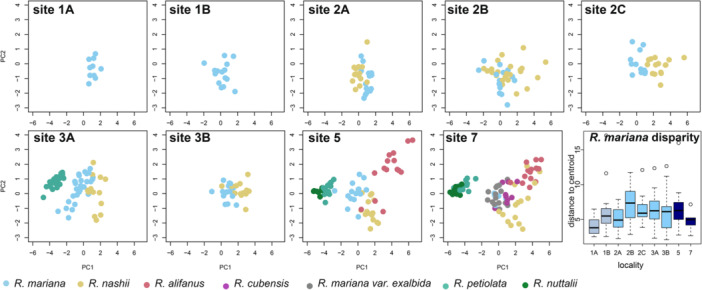
Flower trait space (PCA) and floral disparity (trait variance) for the different study sites (based on flower trait measurements indicated in Figure 1B, C), showing that *R. mariana* occupies the center of the trait space at all study sites, and overlaps partially with large‐flowered *R. nashii* (most commonly co‐occurring) but not with small‐flowered *R. petiolata* (second most commonly co‐occurring). The second small‐flowered species, *R. nuttallii*, occupies a distinct area close to *R. petiolata*, while two other large‐flowered species (*R. cubensis, R. mariana var. exalbida*) overlap substantially with *R. mariana*. The largest‐flowered species, *R. alifanus*, overlaps with *R. nashii*, but is separated from *R. mariana*. Floral disparity (measured as distance to centroid of each locality) of *R. mariana* did not vary consistently with co‐flowering context, and localities with species (partially) overlapping in trait space showed similar disparity as localities 1A and 1B where *R. mariana* occurred on its own.

To establish whether *Rhexia mariana* overlaps in floral trait space with other *Rhexia* species, which increases the possibility of HPT given the shared pollinators, we compared floral functional traits across the seven *Rhexia* species (Figure 6). *Rhexia* species differed significantly in flower morphology across all study sites (R² = 0.698, pseudo‐F ratio = 142.81, p‐value <0.001) and within study sites (Appendix S1: Table S9, S10). 74% of floral trait variation is explained by the first two axes. Flowers of *R. mariana* occupied the center of the floral trait space (Figure 6) and were clearly distinct from the two species that have smaller corollas and small, upright stamens (*R. petiolata*, *R. nuttallii*). The species that co‐flowered with *R. mariana* most commonly, *R. nashii*, clustered next to *R. mariana* and was significantly different at all co‐flowering localities (Appendix S1: Table S9, S10). *Rhexia cubensis* and *R. mariana var. exalbida*, occur at highest co‐flowering intensities, and were the only pairwise comparisons determined to not be significantly different (Appendix S1: Table S10).

To more specifically test whether the different *Rhexia* species overlap in traits critical for pollen transfer, we next compared whether the species differ in the distance between the anther base, where bumblebees grasp *Rhexia* stamens to apply vibrations, and the stigma. Comparing the maximum and minimum anther‐base‐stigma distance across species and localities, we found that anther‐base‐stigma distances of *R. mariana* (maximum: 12.6 mm, minimum: 5.57 mm) were intermediate, and either significantly smaller (most comparisons with *R. alifanus* (max.: 15.6 mm, min.: 9.4 mm), *R. nashii* (max.: 15.9 mm, min.: 6.89 mm)) or larger (compared with *R. petiolata* (max.: 3.98 mm, min.: 2.33), *R. nuttallii* (max.: 2.85 mm, min.: 1.14 mm)), but overlapped with *R. cubensis* (max.: 12.7 mm, min.: 5.74 mm) and *R. mariana var. exalbida* (max.: 12.4 mm, min.: 5.61 mm; Appendix S1: Figure S6, S7; Table S11, S12). Distances between *R. mariana* and *R. nashii* overlapped at locality 2B where we detected the most diverse bee pollinator fauna (Figure 3).

### Floral differentiation and variance of *Rhexia mariana* across co‐flowering contexts

Next, we compared floral traits of *R. mariana* across co‐flowering contexts to explore whether flower trait space occupation differs among co‐flowering intensities. Using PERMANOVAs, we found that flowers of *R. mariana* from low co‐flowering contexts differed significantly in trait space occupation from flowers from high co‐flowering contexts (R² = 0.09, F = 4.39, p < 0.01), while intermediate co‐flowering contexts differed neither from low (R² = 0.01, F = 1.71, p = 0.14) nor high (R² = 0.02, F = 1.55, p = 0.15) co‐flowering contexts (Appendix S1: Figure S8). Flowers of low co‐flowering intensity had significantly larger maximum anther‐base‐stigma distances (13.6 mm on average) than flowers from high (12.1 mm, X² = –2.22, p = 0.04) and intermediate (12.5 mm, X² = –2.15, p = 0.048) co‐flowering intensities. Overall, however, we found strong differentiation among localities (R² = 0.06, F = 7.94, p < 0.01; 41 significant differences out of 45 pairwise comparisons, Appendix S1: Table S13) regardless of co‐flowering contexts.

To test whether high co‐flowering intensities led to lower floral trait variances, potentially indicative of selection for more constrained phenotypes (both under negative or positive pollinator‐mediated plant‐plant interactions, Figure 1), we compared floral disparity of *R. mariana* across contexts and localities. Against expectations, we found the lowest floral trait variances in low co‐flowering contexts (average distance to centroid 5.85), with these being significantly lower than in intermediate (average distance to centroid 7.98) and high (average distance to centroid 6.91) co‐flowering intensities (F = 6.91, df = 2, p = 0.001). There were no significant differences in trait variance between high and intermediate co‐flowering intensities. When comparing trait variances across study localities, we found no significant differences (F = 1.95, df = 8, p = 0.57; Figure 6); also, neither mean differences nor differences in variance were explained by geographic distance among sites (mean: R² = 0.14, p = 0.29, variance: R² = 0.26, p = 0.08).

Calculating community‐weighted dispersion for all floral traits with and without *R. mariana* for the co‐flowering localities showed that corolla diameter and the level of anther pores were more variable when including *R. mariana*, indicating more variable floral displays of *R. mariana* in co‐flowering sites (Appendix S1: Figure S9). In contrast, fit traits such as the horizontal spread of stamens, stigma length and stigma height were less variable when including *R. mariana*, indicating less variation in these traits. All other traits showed comparable dispersion.

## DISCUSSION

Using *Rhexia* as a model, we here combine a macroevolutionary (congeneric) perspective on pollination niche differentiation and floral divergence with an assessment of the impact of community‐level processes on pollinator‐mediated plant‐plant interactions in *Rhexia mariana*. This combination of scales allows for exploring realized ecological interactions within a macroevolutionary framework and may help inform on the role of community‐level processes in shaping macroevolutionary responses. Given that we did not find support for either negative (i.e., competition) or positive (i.e., facilitation) effects of community‐level processes in the pollination of *R. mariana*, we may deduce that present‐day interactions with relatives may be mostly neutral (see also Armbruster and McGuire, 1991; Mesquita‐Neto et al., 2018 for similar results on congeners), and so perhaps played a minor role in the evolution of plant‐pollinator interactions in the lineage. Given that all *Rhexia* species shared the same pollinators, the observed differences in mean floral phenotypes may be more readily interpreted as a by‐product of lineage divergence rather than because of specialization on distinct bee pollinators. In contrast, the increased trait variance of *R. mariana* in co‐flowering sites, particularly brought about by larger variability in corolla diameter and pore height, may have various causes, including selection for variable displays to match different co‐flowering congeners (facilitation), selection for more variable pollen placement on pollinators through variation in the level of anther pores (potentially increasing outcross pollen transfer or genetic diversity of pollen; Dai et al., 2016), or signatures of hybridization. In the following sections, we discuss our results both at the level of community‐level processes, and in the context of a macroevolutionary perspective related to the maintenance of species boundaries in *Rhexia*.

### Pollinator‐mediated plant‐plant interactions in co‐flowering *Rhexia* species

Contrary to expectations under negative interactions (Figure 1), we found co‐flowering *Rhexia* species to largely overlap in pollination niche space, and the same bumblebee species, *Bombus impatiens*, being the most important pollinator of *Rhexia mariana* across a stretch of ~100 kilometers and different habitat types. Although *Bombus impatiens* only showed relatively low constancy on *R. mariana* (assessed at high co‐flowering intensity study locality 7), our assessments of visitation rates and pollination performance did not indicate negative competitive interactions in co‐flowering localities, which would be expected to lead to high pollination niche partitioning and reductions in pollinator diversity and pollination performance at the focal species (Figure 1). At the other extreme, positive facilitative effects through pollinator sharing also could not be confirmed for *Rhexia mariana* in our study. Generally, facilitation is expected to be particularly important when pollinators are scarce and plants are pollen‐limited, so that the benefits of increased pollinator attraction through shared floral display and rewards under high co‐flowering intensities outweigh costs of heterospecific pollen receipt (Bergamo et al., 2020). Comparing stigmatic pollen loads (Appendix S1: Table S6) with preliminary ovule counts (ca. 456 ovules per flower, Dellinger, unpublished data) showed that all but one locality (single‐flowering locality 1B) was pollen‐ limited. This pollen limitation is relatively severe, with an average stigmatic pollen load across sites having the potential of fertilizing only 64% of ovules (and only 36% if excluding site 1B). However, despite this strong pollen limitation and the overall visual similarity of *Rhexia* species (pink corollas, yellow stamens), facilitation through joint pollinator signalling and rewarding could not be confirmed in our study.

In general, strong pollen limitation such as what we detected in *Rhexia mariana* not only increases the probability of facilitative effects among co‐flowering congeners but also increases the potential for pollinator‐mediated selection on floral traits (Koski, 2023). Overall, the co‐flowering *Rhexia* species differed significantly in flower morphology, both in the entire phenotype and in traits critically important in mediating fit with pollinators (Figure 6). This interspecific differentiation is expected particularly if negative interactions (i.e., reproductive interference) shape flower trait divergence (Figure 1). Patterns of trait variance, however, did not match expectations under negative interactions, where reduced trait variance is expected under high co‐flowering intensities and unconstrained, higher variance under low co‐flowering intensities (Figure 1). The rationale behind these expectations is that under high co‐flowering intensity, selection for reduced HPT may be particularly strong so that pollinator‐mediated selection may lead to low trait variance in the focal study species, balancing the focal species on a narrow adaptive ridge (Armbruster et al., 2009; Pérez‐Barrales and Armbruster, 2023). At low co‐flowering intensities, however, the species may be less constrained because there is no risk of congeneric HPT (Jacquemyn and Brys, 2020; Paglia et al., 2023). In our study, we found the opposite to be true, however, with lowest trait variances under low co‐flowering intensity. Such a scenario may be indicative of facilitative effects, in that pollinator scarcity (under low co‐flowering intensity) may lead to selection favoring optimally adapted phenotypes (which may maximize pollen transfer), while higher trait similarity with congeners (achieved through increased variance in corolla diameter in *R. mariana*, matching larger‐flowered *R. nashii* and *R. alifanus* as well as smaller‐flowered *R. petiolata* and *R. nuttallii*) may be beneficial in high co‐flowering intensities (Figure 1). However, given that selection on floral traits may not only stem from pollinators but also from abiotic factors or other processes in the community (Eisen et al., 2020), more detailed studies are needed in the future to explore the drivers of trait variance in *R. mariana*.

Despite efforts to provide a broad and yet detailed assessment of the pollination biology of *Rhexia mariana*, there are several caveats to our study that may obscure effects of co‐flowering. First, our assessments of plant‐pollinator interactions did not span the entire flowering season of *R. mariana and* so cannot capture variation in pollinator‐mediated plant‐plant interactions throughtout the season. Seasonal variation (intra‐annual or interannual) in the availability of pollinators and co‐flowering species may strongly affect interaction patterns and change the effects of selection on flower traits (Cha et al., 2023; Koski, 2023; Pérez‐Barrales et al., 2025). Second, our assessments include relatively few sites (nine), and it is possible that locally high availability of pollinators (e.g., site 1B) masks general patterns that would become more apparent if a broader sample of sites was included. Similarly, the relative geographic vicinity of some sites (e.g., 1B and 3B) may blur differences in visitation rates in that bumblebees may fly between geographically adjacent sites (Knight et al., 2005). Given that visitation rates seemed unaffected by geographic distance (i.e., no increase in visitation rates among closer sites), this problem may be small in our assessment. Third, pollinator‐mediated plant‐plant interactions may be strongly affected by the relative densities of co‐flowering species (Eisen et al., 2020), an aspect which we did not assess in our study. *Rhexia mariana* was generally the most common species across sites and most evenly distributed, and was locally interspersed by more dense patches of, for example, *R. nashii* and *R. alifanus*. It is therefore possible that small‐scale patterns of competition or facilitation exist within sites, which remain undetected here. Related to this, at least the common species *R. mariana*, *R. nashii*, and *R. petiolata* may occur also between our study sites, which may affect local‐scale pollinator abundances beyond the scope of our co‐flowering contexts. Fourth, the somewhat surprising result that low co‐flowering intenstiy showed lowest floral trait variances, but that no such pattern was detected among populations, may stem from the fact that we only measured 15 *R. mariana* flowers per study site. Measuring a larger number of flowers across more sites would be required to establish whether indeed, trait variance increases with co‐flowering intensity. Finally, and perhaps most importantly, we only assessed pollination performance (pollen removal and deposition) here, and, given the general similarity of *Rhexia* pollen grains, we were unable to differentiate conspecific from heterospecific congeneric pollen. Assessing pollen‐tube growth is commonly used as a method for distinguishing con‐ from heterospecific pollen transfer when pollen grains cannot be easily identified (e.g., Albor, García‐Franco, et al., 2019). This approach is unfeasible in *Rhexia* given that ovaries hold more than 400 ovules, and individual pollen tubes cannot be distinguished in the slim style. Furthermore, preliminary crossing experiments have shown that species are interfertile (Dellinger, unpublished data), so that pollen tube growth itself may not be a reliable indicator of conspecific pollen deposition. Simulating heterospecific pollen deposition through manipulative crossing experiments may provide an alternative in the future to study the impact of HPD, and to assess whether co‐flowering affects other aspects of reproduction such as seed set, seed quality and seedling viability of *R. mariana* (Hernández‐Moreira et al., 2023; Hao et al., 2023). It is possible, for example, that tolerance to HPD differs among co‐flowering contexts, and that *R. mariana* from high co‐flowering intesity is more tolerant of HPD than from low co‐flowering intensity (compare Hernández‐Moreira et al., 2023). Including such assessments in the future will be essential to clarify whether co‐flowering does indeed have neutral rather than positive or negative effects in *R. mariana*.

### Pollinator sharing and reproductive barriers in co‐flowering *Rhexia* species

The high pollinator sharing of *Rhexia* reported here begs the question of how species boundaries are maintained in the genus. Two of the reproductive barriers commonly considered as most important in plants, geographical isolation through allopatry, and temporal isolation through differentiated phenology (Baack et al., 2015), are non‐existent in *Rhexia*. Our assessments of co‐flowering localities further indicate that ethological isolation (attraction of different pollinators; Armbruster, 2014) is also weak, as might be expected given the overall similar floral display of the *Rhexia* species assessed here. Noteably, the *Rhexia* species with the most differentiated floral phenotype, *Rhexia lutea* with yellow petals and stamens, is also the only species that has a differentiated phenology (flowering before the other *Rhexia* species in Florida; Kral and Bostick, 1969). *Rhexia lutea* also occurred at our study locality 7 but was fruiting at peak flowering times of the other species. The pollinators of *Rhexia lutea* are currently unknown. It therefore remains unclear whether the combined phenotypic and phenological differentiation of *R. lutea* is the result of pollinator‐mediated selection (e.g., due to competition during secondary contact), or an adaptation to the non‐*Rhexia* co‐flowering community (e.g., facilitation by other yellow‐flowering genera such as *Hypericum* (Hypericaceae) and *Ludwigia* (Onagraceae) earlier in the season).

A reproductive barrier identified as important in reducing heterospecific pollen transfer in co‐flowering congeners is differential pollen placement on the same pollinator (Armbruster et al., 1994; Muchhala and Potts, 2007; Eisen and Geber, 2018). With the diffuse scattering of pollen grains across the bodies of bumblebees’ bodies in *Rhexia*, in addition to frequent readjustments of buzzing positions and thus repeated stigma contact with different body parts, such refined and differentiated pollen placement seems unlikely, increasing the probability of HPT. It is possible, however, that the actual rates of HPT are low. Although we were unable to assess HPT directly given that pollen grains in *Rhexia* are all very similar and fluorescent dyes cannot be applied accurately to poricidal anthers, we may deduct some rationales from our assessments of floral constancy and pollinator behavior. The majority (85%) of individuals of *B. impatiens* showed at least one interspecific transition with potential for HPT. However, our transition rate matrix showed that most visits (72%) were intraspecific, meaning that a single bumblebee individual may visit several flowers of *Rhexia mariana* in a row, before switching to another species. Now, it is unclear how much pollen gets transferred in a single visit, but it is possible that the relative frequency of intraspecific visits is sufficient to assure high conspecific pollen receipt (Tong and Huang, 2016). Second, the generally diffuse pollen placement during buzz‐pollination in *Rhexia* may create a diffuse intra‐ and interspecific pollen landscape on the bee's body (Minnaar et al., 2019), perhaps faciliated by the increased variance in the level of anther pores in *R. mariana* in co‐flowering sites (Appendix S1: Figure S9). While interspecific pollen will likely be dispersed between intraspecific pollen, the net load of interspecific pollen at any single spot on the bumblebee's body (which might be touched by the punctiform stigma) might be relatively low. Molecular techniques such as pollen DNA metabarcoding with markers specifically designed to differentiate the different *Rhexia* species may help resolve the composition of pollen loads carried by bumblebees pollinating *Rhexia* (James et al., 2022).

When pre‐pollination reproductive isolation is weak, post‐pollination and post‐zygotic reproductive barriers (e.g., no or lower germination rates of hybrid seeds, lower hybrid seedling viability, and fertility) are expected to be highly important in maintaining species boundaries. While molecular data point towards reticulate evolution and a major role of hybridization in *Rhexia* (Judd and Ionta, 2023), the mechanisms and relative contribution of post‐pollination reproductive barriers in *Rhexia* remain largely unexplored. Hand‐pollination experiments of several species have shown abundant formation of fruits after interspecific crosses (Kral and Bostick, 1969), a result that we could confirm in a preliminary set of crosses with *R. mariana* as recipient and *R. alifanus*, *R. nashii*, and *R. petiolata* as donors in locality 7 (Dellinger, unpublished). However, Kral and Bostick (1969) also reported frequent seed abortion or reduced fitness of hybrids in crosses with *R. mariana*, perhaps lowering their likelihood to establish in natural populations. Notably, Kral and Bostick (1969) also pointed out that hybrids of *R. mariana* with other species often take the shape of the respective interspecific pollen donor and therefore may not be identifiable as hybrids morphologically. This may be complicated by the fact that several *Rhexia* species, including *Rhexia mariana*, *R. nashii*, and *R. cubensis*, have polyploid series, which are usually not distinct from each other morphologically. While polyploidization seems to be preceded by hybridization in *Rhexia* (e.g., *R. nashii* is likely an allopolyploid formed through hybridization of *R. mariana* and *R. virginica;* Ionta et al., 2007), polyploidy may also serve as a reproductive barrier at the intraspecific level (as diploid and tetraploid *R. mariana* cannot interbreed; Kral and Bostick, 1969) and potentially also the interspecific level (Castro et al., 2020). Establishing, for example, whether *R. mariana* is more likely to be diploid when co‐flowering with common *R. nashii* (no diploids known) than when flowering on its own, would be a first step towards evaluating whether polyploidy in *Rhexia* may act as a reproductive isolating mechanism or instead fosters pollinator inconstancy (see Schmickl et al., 2024), and ultimately hybridization (Ionta et al., 2007).

## CONCLUSIONS: LEARNING FROM *RHEXIA* ABOUT FLOWER EVOLUTION IN MELASTOMATACEAE?

At least at the time of our study, interactions between buzzing bees and *Rhexia mariana* flowers were stable across multiple co‐flowering contexts and localities in Florida. Finding the same buzzing bee pollinators and neutral rather than positive or negative effects of co‐flowering is particularly interesting in the context of Melastomataceae having the largest radiation of buzz‐pollinated flowers, with multiple, florally disparate species commonly co‐flowering (Kopper et al., 2024). However, our results suggest that pollinator‐mediated competition among co‐flowering relatives may not have been the primary driver of floral differentiation in the group (i.e., in adaptation to distinct bees). With the often‐diffuse scattering of pollen grains during buzz‐pollination, however, the question arises whether specialization on distinct bees is likely at all. Other studies on buzz‐pollination have shown that any bee large enough to bridge the gap between the anther base where vibrations are applied, and the stigma, may act as effective pollinator (Mesquita‐Neto et al., 2021, Mesquita‐Neto and Schlindwein, 2025; Dellinger et al., 2023). This might indicate that bee pollinators are relatively exchangeable across communties and species as long as they are large enough to bridge the anther‐stigma distance (Larson and Barrett, 1999). To further explore how co‐flowering community‐level processes link back to (and perhaps explain) macroevolutionary differences among species, it will be essential to replicate our study in different Melastomataceae communities that have higher phylogenetic and morphological variability, and to experimentally manipulate the density and identity of co‐flowering relatives (Ha and Ivey, 2017).

## AUTHOR CONTRIBUTIONS

A.S.D. designed the project, planned fieldwork, collected data, analysed the data and wrote the initial manuscript draft. K.G., K.D.P.A., and V.C.W. counted pollen grains, K.G. reviewed pollinator videos, analysed data and helped write the manuscript draft, and A.K. helped with field data collection and mounting of the bees. All authors commented on later versions of the manuscript.

## Supporting information


**Appendix S1.** Supplemental figures and tables.
**Figure S1.** Species richness of *Rhexia* across the US based on pruned data obtained from GBIF.
**Figure S2.** Molecular phylogeny of the genus *Rhexia*.
**Figure S3.** Pollinator community of *Rhexia mariana* summarized by co‐flowering context.
**Figure S4.** Visit time and duration of the different bee pollinators according to co‐flowering context on *R. mariana*.
**Figure S5.** Neither male pollination performance (number of pollen remaining in stamens) nor female pollination performance (number of pollen grains deposited on stigma) was explained by visitation rates or co‐flowering context in *Rhexia mariana*.
**Table S1.** Conceptual overview of questions which can be addressed when performing”community”‐level studies (i.e., co‐flowering species) within a macroevolutionary entity (i.e., congeneric).
**Table S2.** The nine study localities, geographic coordinates, *Rhexia* species composition and study dates.
**Table S3.** Distances (km) between study sites.
**Table S4.** Total duration of visitor observations (in minutes) and median visitation rate (VR).
**Table S5.** Shannon index of pollinator diversity at the different study sites and on *R. mariana* (focal species).
**Table S6.** Pollination performance estimated through median (SD) amount of pollen grains. remaining in stamens at the end of anthesis, and pollen grains deposited on stigmas of *Rhexia mariana* across localities.
**Table S7.** Results of GLMM on pollen remaining and pollen deposited across co‐flowering contexts.
**Table S8.** Results of GLMM on pollen remaining and pollen deposited across study sites.
**Table S9.** Results of PERMANOVA on floral traits for each study site with co‐flowering *Rhexia* species.
**Table S10.** Pairwise comparisons of floral traits of all co‐flowering species.
**Table S11.** Kruskal‐Wallis ANOVA on maximum and minimum distance between stamen base and stigma.
**Table S12.** Dunn‐test results on pairwise differences among species in multi‐species sites 3A, 5 and 7.
**Table S13.** Pairwise differences in floral trait space occupation among localities of *R. mariana*.

## Data Availability

All datasets analysed in this study are available on the public repository Phaidra: Website: https://phaidra.univie.ac.at/o:2091633.
