# Supplementary material for: Co‐flowering with congeners does not affect buzz‐pollinator specialization and pollination performance in Rhexia mariana, but does affect floral trait variance
Source: Am J Bot. 2025 Oct 28;112(11):e70119. doi: 10.1002/ajb2.70119 (PMC12640474; doi:10.1002/ajb2.70119)
Supplement: Supplementary file 1 — Appendix S1. Supplemental figures and tables. Figure S1. Species richness of Rhexia across the US based on pruned data obtained from GBIF. Figure S2. Molecular phylogeny of the genus Rhexia. Figure S3. Pollinator community of Rhexia mariana summarized by co‐flowering context. Figure S4. Visit time and duration of the different bee pollinators according to co‐flowering context on R. mariana. Figure S5. Neither male pollination performance (number of pollen remaining in stamens) nor female pollination performance (number of pollen grains deposited on stigma) was explained by visitation rates or co‐flowering context in Rhexia mariana. Table S1. Conceptual overview of questions which can be addressed when performing”community”‐level studies (i.e., co‐flowering species) within a macroevolutionary entity (i.e., congeneric). Table S2. The nine study localities, geographic coordinates, Rhexia species composition and study dates. Table S3. Distances (km) between study sites. Table S4. Total duration of visitor observations (in minutes) and median visitation rate (VR). Table S5. Shannon index of pollinator diversity at the different study sites and on R. mariana (focal species). Table S6. Pollination performance estimated through median (SD) amount of pollen grains. remaining in stamens at the end of anthesis, and pollen grains deposited on stigmas of Rhexia mariana across localities. Table S7. Results of GLMM on pollen remaining and pollen deposited across co‐flowering contexts. Table S8. Results of GLMM on pollen remaining and pollen deposited across study sites. Table S9. Results of PERMANOVA on floral traits for each study site with co‐flowering Rhexia species. Table S10. Pairwise comparisons of floral traits of all co‐flowering species. Table S11. Kruskal‐Wallis ANOVA on maximum and minimum distance between stamen base and stigma. Table S12. Dunn‐test results on pairwise differences among species in multi‐species sites 3A, 5 and 7. Table S13. Pairwise differences in flor [file AJB2-112-e70119-s001.docx]

**Supplementary Material for****:**

**Co-flowering with congeners does not affect pollination niche partitioning and pollination performance in *Rhexia mariana,* but floral trait variance**


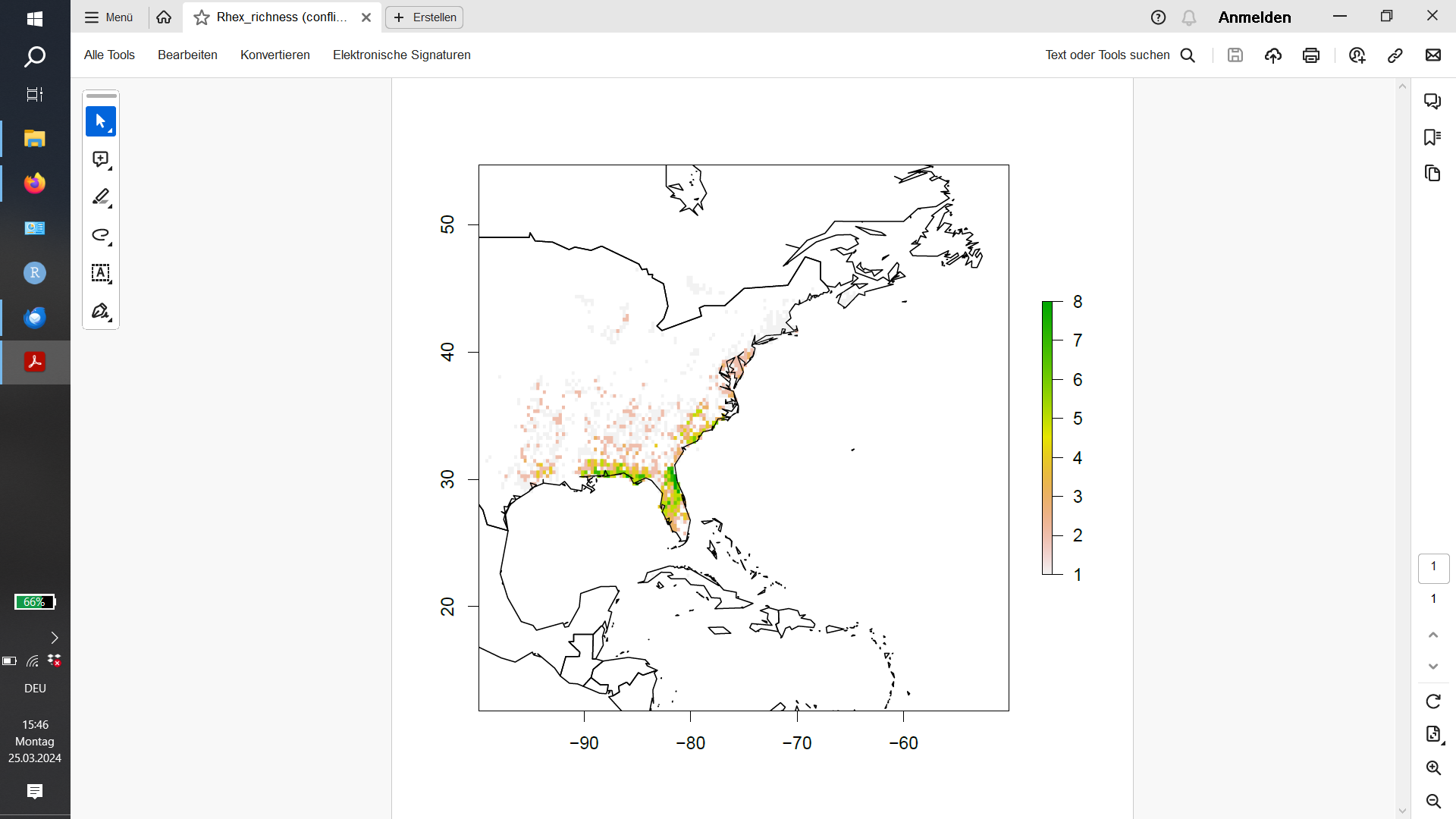


**Figure S1. Species richness of *Rhexia* across the US based on pruned data obtained from gbif**. The Southeastern US holds the highest species richness, with our study locality in Gainesville, Florida featuring up to eight co-occurring *Rhexia* species. Species richness was calculated in 1x1 kilometer grid cells.

Global Biodiversity Information Facility, GBIF.org (13 October 2025) GBIF

occurrence download https://doi.org/10.15468/dl.yfvue2


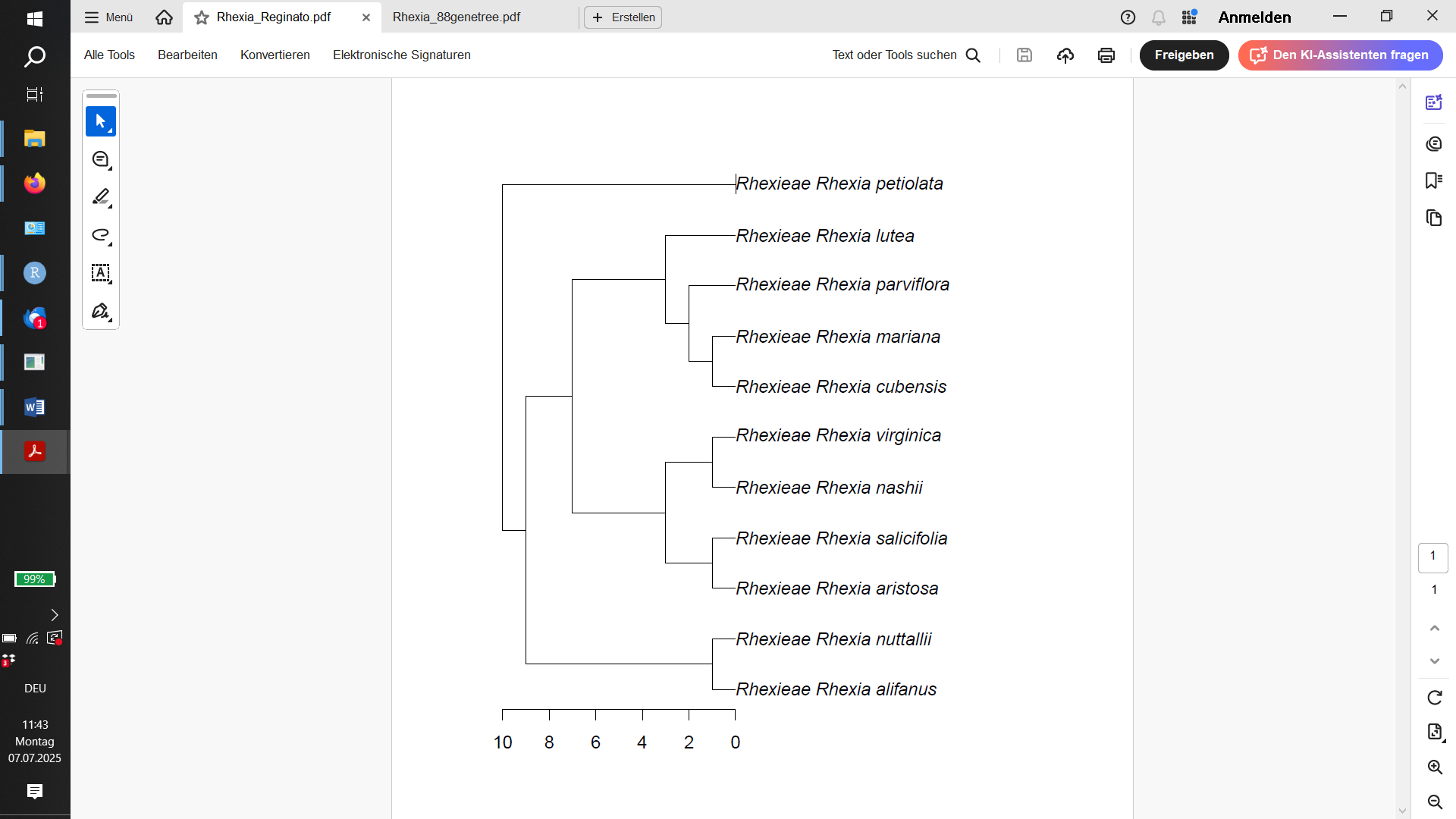


**Figure S2. Molecular phylogeny of the genus *Rhexia***, pruned from the most recent molecular phylogeny of Melastomataceae by Reginato et al. (2022), consensus-tree based off Penneys et al. (2022). Based on time-calibrations performed in Reginato et al. (2022), the crown age of *Rhexia* is approximately 10 mya, while crown ages for sister species pairs are only about 1 mya. The closest relative of *R. mariana* seems to be *R. cubensis* (co-flowering in site 5 and 7), forming a clade together with *R. parviflora* and *R. lutea* (not considered in this study). Our study further included *R. nashii* (in a separte clade) and *R. alifanus* and *R. nuttallii* (forming a clade sister to the remaining *Rhexias*, except for *R. petiolata*, which is here reconstructed as sister to all other *Rhexias*). Note that these are preliminary phylogenetic treatments of the genus and that sampling multiple accessions per species may yield different infrageneric relationships; there is also evidence for reticulate evolution and hybridization, and other treatments show e.g., *Rhexia nuttallii* and *R. petiolata* as most closely related and sister to *R. alifanus*, with the three species together being sister to all other *Rhexia* (compare Ionta et al. 2007).

**Table S1.** **Conceptual overview of questions which can be addressed when performing ”community”-level studies (i.e., co-flowering species) within a macroevolutionary entity (i.e., congeneric).** We have here explicitly included the data and some of the questions addressed by our study to help readers navigate through our manuscript.

| **level** | **data** | **question** |
| --- | --- | --- |
| macroevolutionary | pollinator composition | Are the different *Rhexia* species specialized on different bees? |
| macroevolutionary | pollinator constancy | Do bees show constancy on *Rhexia mariana* or switch among species? |
| macroevolutionary | diurnal pollinator stratification | Do bees visit different *Rhexia* species at different times of day? |
| macroevolutionary | mean floral traits | Are the different *Rhexia* species significantly differentiated morphologically? |
| community-level | pollinator composition | Is *R. mariana* specialized to a more narrow pollinator guild at multi-species sites? |
| community-level | pollinator visitation rates | Do visitation rates to *R. mariana* differ across localities? |
| community-level | male pollination performance | Is male pollination performance of *R. mariana* lower at multi-species sites (competition)? |
| community-level | female pollination performance | Is female pollination performance of *R. mariana* lower at multi-species sites (competition)? |
| community-level | mean floral traits *R. mariana* | Are flowers of *R. mariana* in high co-flowering contexts more differentiated than in low co-flowering contexts? |
| community-level | floral trait variances *R. mariana* | Are flowers of *R. mariana* in high co-flowering contexts less variable and more specialized? |

**Table S2. The nine study localities, geographic coordinates, *Rhexia* species composition and study dates.**

| **site** | **context** | **locality** | **coordinates** | **species composition** | **study dates** |
| --- | --- | --- | --- | --- | --- |
| 1A | Low | Watermelon Park | 29.577209, -82.615578 | *R. mariana* | 20220704, 20220705 |
| 1B | Low | Tucawilla Preserve | 29.501121, -82.270115 | *R. mariana* | 20220715 |
| 2A | Intermediate | SW Country Road 346 | 29.499326, -82.420955 | *R. mariana, R. nashii* | 20220717 |
| 2B | Intermediate | 375W Thrasher Drive (private garden) | 29.446146, -82.644152 | *R. mariana, R. nashii* | 20220707, 20220712 |
| 2C | Intermediate | Prairie Creek | 29.595971, -82.228616 | *R. mariana, R. nashii* | 20220710 |
| 3A | Intermediate | Flatwood Preserve and roadside | 29.568728, -82.192419 | *R. mariana, R. nashii, R. petiolata* | 20220706, 20220709 |
| 3B | Intermediate | roadside close to Prairie Creek | 29.5248800, -82.2761620 | *R. mariana, R. nashii, R. petiolata* | 20220714 |
| 5 | High | Etoniah State Forest | 29.800558, -81.820316 | *R. mariana, R. nuttallii, R. alifanus, R. nashii, R. petiolata* | 20220711, 20220713 |
| 7 | high | Jennings State Forest | 30.164566, -81.935360 | *R. mariana, R mariana var. exalbida, R. alifanus, R. petiolata, R. nuttallii, R. cubensis, R. nashii,* | 20220708, 20220716, 20220720 |

**Table S3. Distances (km) between study sites.**

|  | 1B | 2A | 2B | 2C | 3A | 3B | 5 | 7 |
| --- | --- | --- | --- | --- | --- | --- | --- | --- |
| 1A | 34.53 | 20.75 | 14.79 | 37.55 | 41.01 | 33.40 | 80.85 | 92.51 |
| 1B |  | 14.63 | 36.79 | 11.26 | 10.62 | 2.70 | 54.76 | 80.34 |
| 2A |  |  | 22.44 | 21.50 | 23.45 | 14.32 | 67.06 | 87.41 |
| 2B |  |  |  | 43.57 | 45.86 | 36.74 | 88.93 | 105.06 |
| 2C |  |  |  |  | 4.63 | 9.13 | 45.56 | 69.10 |
| 3A |  |  |  |  |  | 9.46 | 44.24 | 70.56 |
| 3B |  |  |  |  |  |  | 53.68 | 78.18 |
| 5 |  |  |  |  |  |  |  | 41.85 |

**Table S4. Total duration of visitor observations** (in minutes) **and median visitation rate (VR)** of *Rhexia mariana* per study locality and across all species per locality.

| **site** | **context** | **duration** | **median VR *R. mariana*** | **median VR across species** |  |
| --- | --- | --- | --- | --- | --- |
| 1A | Low | 413 | 0.153 | 0.153 | |
| 1B | Low | 502 | 0.733 | 0.733 | |
| 2A | Interm. | 170 | 0.300 | 0.300 | |
| 2B | Interm. | 837 | 0.350 | 0.100 | |
| 2C | Interm. | 298 | 0.393 | 0.500 | |
| 3A | Interm. | 1195 | 0.396 | 0.733 | |
| 3B | Interm. | 356 | 0.482 | 0.813 | |
| 5 | High | 753 | 0.188 | 0.125 | |
| 7 | High | 1084 | 0.611 | 0.722 | |


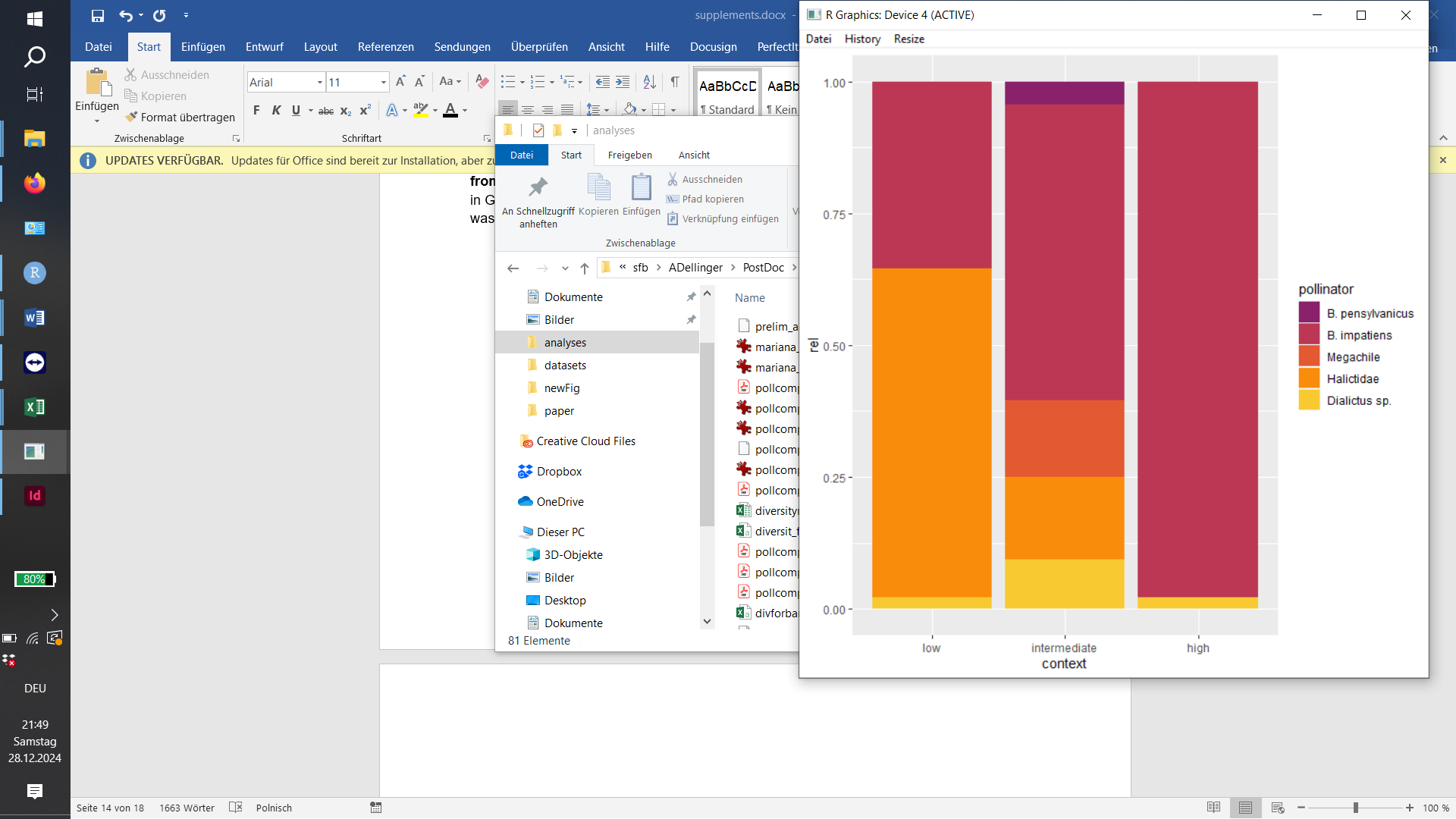


**Figure S3. Pollinator community of *Rhexia mariana* summarized by co-flowering context**, indicating higher pollinator diversity at intermediate co-flowering contexts (note that more study localities were included in this category).


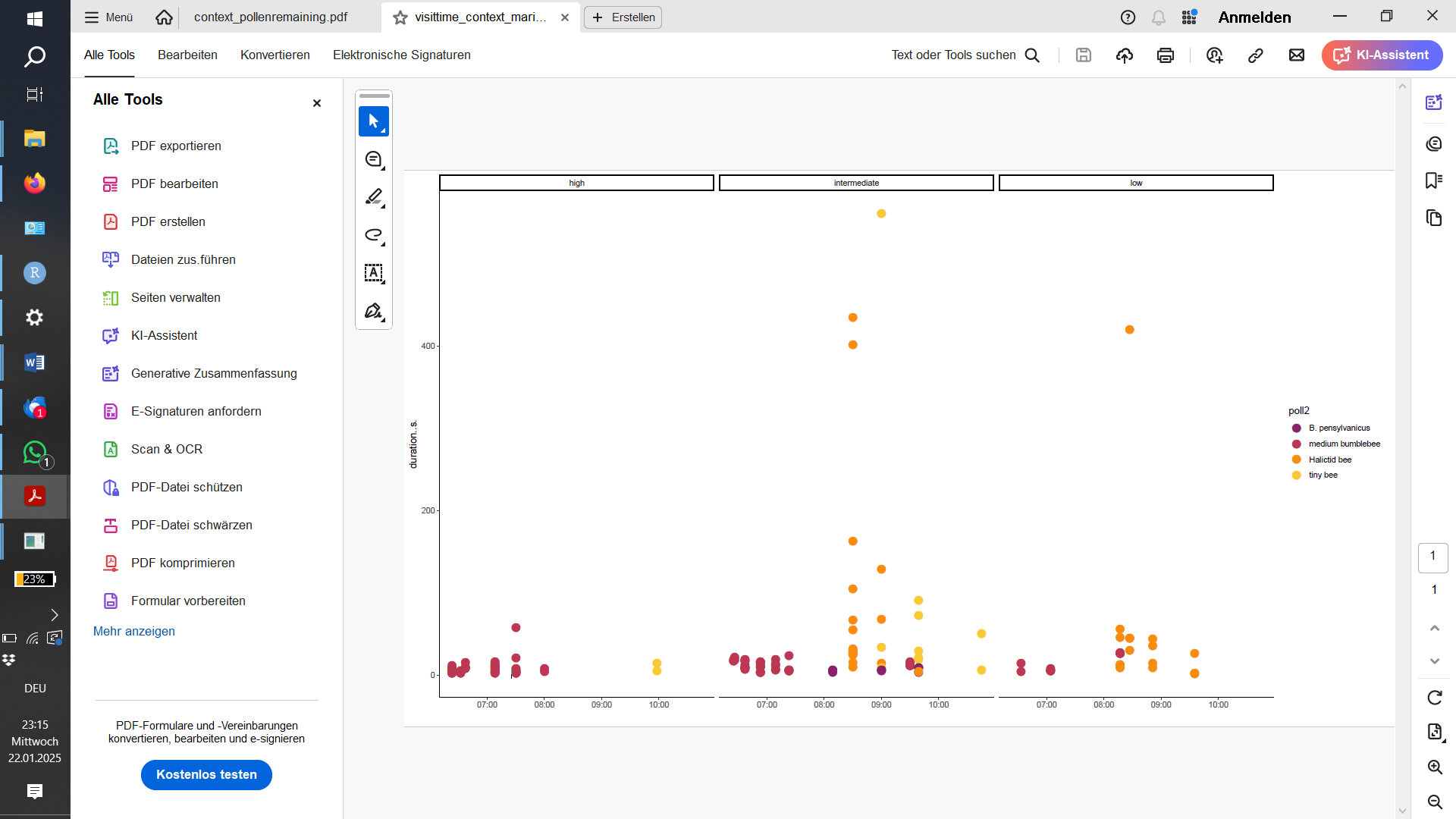


**Figure S4. Visit time and duration of the different bee pollinators according to co-flowering context on *R. mariana***. There was no diurnal stratification in the pollinator community according to co-flowering context.

**Table S5. Shannon index of pollinator diversity at the different study sites and on *R. mariana* (focal species).**

| **site** | **Shannon index - site** | **Shannon index – *R. mariana*** |
| --- | --- | --- |
| 1A | 0.689 | 0.687 |
| 1B | 0.659 | 0.659 |
| 2A | 1.074 | 1.061 |
| 2B | 1.242 | 0.692 |
| 2C | 0.213 | 0.096 |
| 3A | 1.335 | 0.793 |
| 3B | 0.476 | 0.661 |
| 5 | 0.927 | 0.016 |
| 7 | 0.442 | 0.218 |


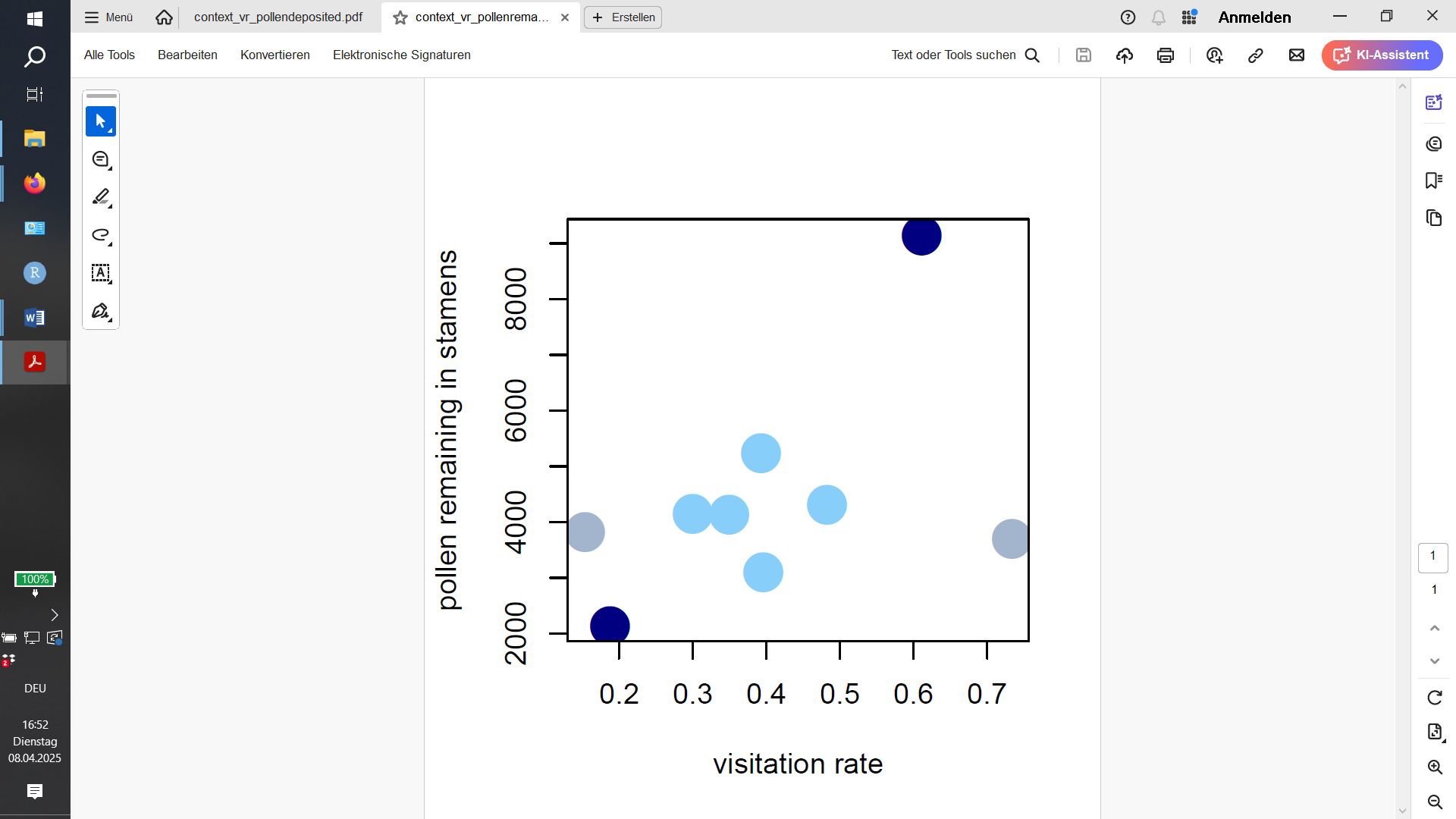

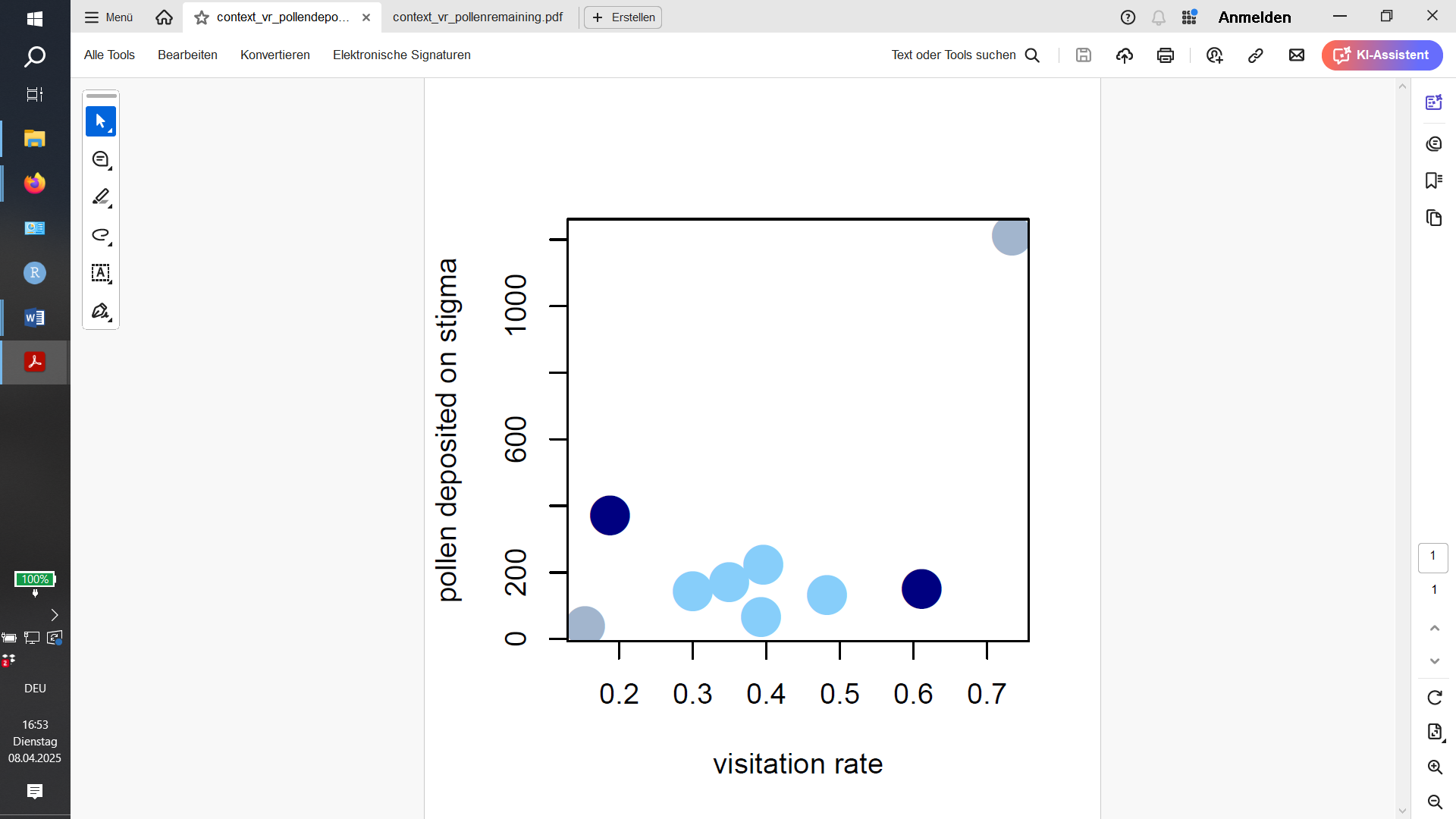


**Figure S5. Neither male pollination performance (number of pollen remaining in stamens) nor female pollination performance (number of pollen grains deposited on stigma) was explained by visitation rates or co-flowering context** in *Rhexia mariana*. A negative relationship was expected for male performance, and a positive relationship for female performance. These results indicate that a substantial proportion of floral visitors (i.e., small halictid bees) are inefficient pollinators and do not contribute to successful pollination.

**Table S6. Pollination performance estimated through median (sd) amount of pollen grains remaining in stamens at the end of anthesis, and pollen grains deposited on stigmas of *Rhexia mariana* across localities.**

| **Pollen remaining in stamens** | | |
| --- | --- | --- |
| **locality** | **median** | **sd** |
| 1A | 4733.0 | 7008.894 |
| 1B | 3291.5 | 1913.247 |
| 2A | 4160.0 | 8641.253 |
| 2B | 4147.0 | 5455.400 |
| 2C | 5238.0 | 3964.651 |
| 3A | 3103.0 | 2634.064 |
| 3B | 4313.0 | 1521.292 |
| 5 | 2143.0 | 3079.696 |
| 7 | 9152.0 | 2962.482 |
|  |  |  |
| **Pollen deposited on stigma** | | |
| **locality** | **median** | **sd** |
| 1A | 40.0 | 106.3731 |
| 1B | 1326.5 | 907.5781 |
| 2A | 144.0 | 147.6257 |
| 2B | 172.0 | 351.6262 |
| 2C | 67.0 | 101.1859 |
| 3A | 224.0 | 217.5438 |
| 3B | 132.0 | 111.3631 |
| 5 | 373.0 | 833.6829 |
| 7 | 151.5 | 204.5707 |

**Table S7. Results of GLMM on pollen remaining and pollen deposited across co-flowering contexts**, with high co-flowering context used as reference level.

| **Pollen remaining** |  |  |  |  |
| --- | --- | --- | --- | --- |
| **context** | **Estimate** | **std_error** | **z-value** | **p-value** |
| (Intercept) | 8.56749 | 0.27596 | 31.046 | <2e-16 |
| intermediate | 0.09973 | 0.32492 | 0.307 | 0.759 |
| low | 0.06213 | 0.39146 | 0.159 | 0.874 |
|  |  |  |  |  |
| **Pollen deposition** |  |  |  |  |
| **context** | **Estimate** | **std_error** | **z-value** | **p-value** |
| (Intercept) | 6.0852 | 0.5579 | 10.907 | <2e-16 |
| intermediate | -0.8211 | 0.6573 | -1.249 | 0.212 |
| low | -0.2539 | 0.7907 | -0.321 | 0.748 |

**Table S8. Results of GLMM on pollen remaining and pollen deposited across study sites,** with study site 1A (single-flowering locality) used as reference value; significantly different study sites are highlighted in bolt. Note that significant differences in pollen removal do not necessarily go along with significant differences in pollen deposition, suggesting variability in pollen transfer rates between study localities.

| **Pollen remaining** | |  |  |  |
| --- | --- | --- | --- | --- |
| **site** | **Estimate** | **std-error** | **z-value** | **p-value** |
| (Intercept) | 8.9846 | 0.1695 | 53.00 | <0.001 |
| **Site 1B** | **-0.7571** | **0.2504** | **-3.02** | **0.00250** |
| Site 2A | 0.1064 | 0.2316 | 0.46 | 0.64593 |
| Site 2B | -0.2210 | 0.2316 | -0.95 | 0.33999 |
| Site 2C | -0.2450 | 0.2354 | -1.04 | 0.29803 |
| **Site 3A** | **-0.7424** | **0.2316** | **-3.21** | **0.00135** |
| **Site 3B** | **-0.5461** | **0.2316** | **-2.36** | **0.01839** |
| **Site 5** | **-1.0052** | **0.2397** | **-4.19** | **<0.001** |
| Site 7 | 0.1408 | 0.2447 | 0.58 | 0.56513 |
|  |  |  |  |  |
| **pollen deposited** | |  |  |  |
| **site** | **Estimate** | **Std-error** | **z-value** | **p-value** |
| (Intercept) | 4.3328 | 0.3041 | 14.248 | <0.001 |
| **Site 1B** | **2.8874** | **0.4492** | **6.428** | **<0.001** |
| Site 2A | 0.8144 | 0.4155 | 1.960 | 0.05000 |
| **Site 2B** | **1.2821** | **0.4155** | **3.086** | **0.00203** |
| Site 2C | 0.2943 | 0.4223 | 0.697 | 0.48586 |
| **Site 3A** | **1.3415** | **0.4155** | **3.229** | **0.00124** |
| Site 3B | 0.7248 | 0.4155 | 1.744 | 0.08109 |
| **Site 5** | **2.3331** | **0.4301** | **5.425** | **<0.001** |
| **Site 7** | **1.0658** | **0.4389** | **2.428** | **0.01518** |

**Table S9. Results of PERMANOVA on floral traits for each study site** with co-flowering *Rhexia* species.

| study site | R² | F | p-value |
| --- | --- | --- | --- |
| 2A | 0.269 | 10.31 | 0.001 |
| 2B | 0.464 | 31.13 | 0.001 |
| 3A | 0.598 | 37.24 | 0.001 |
| 3B | 0.596 | 41.24 | 0.001 |
| 5 | 0.825 | 74.26 | 0.001 |
| 7 | 0.855 | 80.89 | 0.001 |

**Table S10. Pairwise comparisons of floral traits of all co-flowering species** for three study sites with more than two species (sites 3A, 5, 7). Non-significant differences at site 7 are highlighted in bold.

| **pairs** |  |  | **Sums of sqs** | **F value** | **R²** | **p adjusted** |
| --- | --- | --- | --- | --- | --- | --- |
| **site 3A** |  |  |  |  |  |  |
| *mariana* | *vs* | *petiolata* | 3073.122 | 83.36842 | 0.6068965 | 0.003 |
| *mariana* | *vs* | *nashii* | 3700.411 | 71.09686 | 0.6286369 | 0.003 |
| *petiolata* | *vs* | *nashii* | 6612.195 | 172.12608 | 0.8114329 | 0.003 |
| **site 5** |  |  |  |  |  |  |
| *nashii* | *vs* | *mariana* | 2988.2453 | 46.41030 | 0.6237080 | 0.01 |
| *nashii* | *vs* | *petiolata* | 7421.9906 | 170.99193 | 0.8592908 | 0.01 |
| *nashii* | *vs* | *nutalli* | 8333.2546 | 144.94925 | 0.8734553 | 0.01 |
| *nashii* | *vs* | *alifanus* | 996.7459 | 10.73756 | 0.2771873 | 0.01 |
| *mariana* | *vs* | *petiolata* | 1725.9160 | 49.55420 | 0.6389622 | 0.01 |
| *mariana* | *vs* | *nutalli* | 2481.5599 | 53.88217 | 0.7195594 | 0.01 |
| *mariana* | *vs* | *alifanus* | 3329.2027 | 39.51513 | 0.5852782 | 0.01 |
| *petiolata* | *vs* | *nutalli* | 479.9678 | 26.54799 | 0.5583409 | 0.01 |
| *petiolata* | *vs* | *alifanus* | 8242.1691 | 130.27123 | 0.8230885 | 0.01 |
| *nutalli* | *vs* | *alifanus* | 8899.6747 | 105.97892 | 0.8346182 | 0.01 |
| **site 7** |  |  |  |  |  |  |
| *alifanus* | *vs* | *cubensis* | 846.1433 | 10.910744 | 0.33152529 | 0.021 |
| *alifanus* | *vs* | *mariana* | 1869.8062 | 26.107075 | 0.59190221 | 0.021 |
| *alifanus* | *vs* | *nashii* | 868.0426 | 10.814563 | 0.27862128 | 0.021 |
| *alifanus* | *vs* | *petiolata* | 9293.7188 | 171.231897 | 0.85946026 | 0.021 |
| *alifanus* | *vs* | *white* | 4432.7173 | 70.750680 | 0.71645765 | 0.021 |
| *alifanus* | *vs* | *nutalli* | 13398.5807 | 265.328795 | 0.90454398 | 0.021 |
| ***cubensis*** | ***vs*** | ***mariana*** | **515.1927** | **9.175030** | **0.43329479** | **0.063** |
| *cubensis* | *vs* | *nashii* | 654.5370 | 8.822889 | 0.28624471 | 0.021 |
| *cubensis* | *vs* | *petiolata* | 3543.3067 | 86.196130 | 0.79666555 | 0.021 |
| *cubensis* | *vs* | *white* | 1281.2991 | 24.750243 | 0.52941421 | 0.021 |
| *cubensis* | *vs* | *nutalli* | 5840.9816 | 160.910196 | 0.87972240 | 0.021 |
| *mariana* | *vs* | *nashii* | 1521.8136 | 22.542809 | 0.55602484 | 0.021 |
| *mariana* | *vs* | *petiolata* | 1149.3337 | 42.445086 | 0.70220904 | 0.021 |
| ***mariana*** | ***vs*** | ***white*** | **29.5921** | **0.737791** | **0.03937449** | **1.000** |
| *mariana* | *vs* | *nutalli* | 1844.7985 | 87.011137 | 0.82858961 | 0.021 |
| *nashii* | *vs* | *petiolata* | 8188.9855 | 158.604173 | 0.84994976 | 0.021 |
| *nashii* | *vs* | *white* | 3635.9938 | 60.591204 | 0.68394153 | 0.021 |
| *nashii* | *vs* | *nutalli* | 12092.0156 | 252.685640 | 0.90024427 | 0.021 |
| *petiolata* | *vs* | *white* | 2306.2714 | 67.795301 | 0.70771009 | 0.021 |
| *petiolata* | *vs* | *nutalli* | 608.9759 | 27.853492 | 0.49868847 | 0.021 |


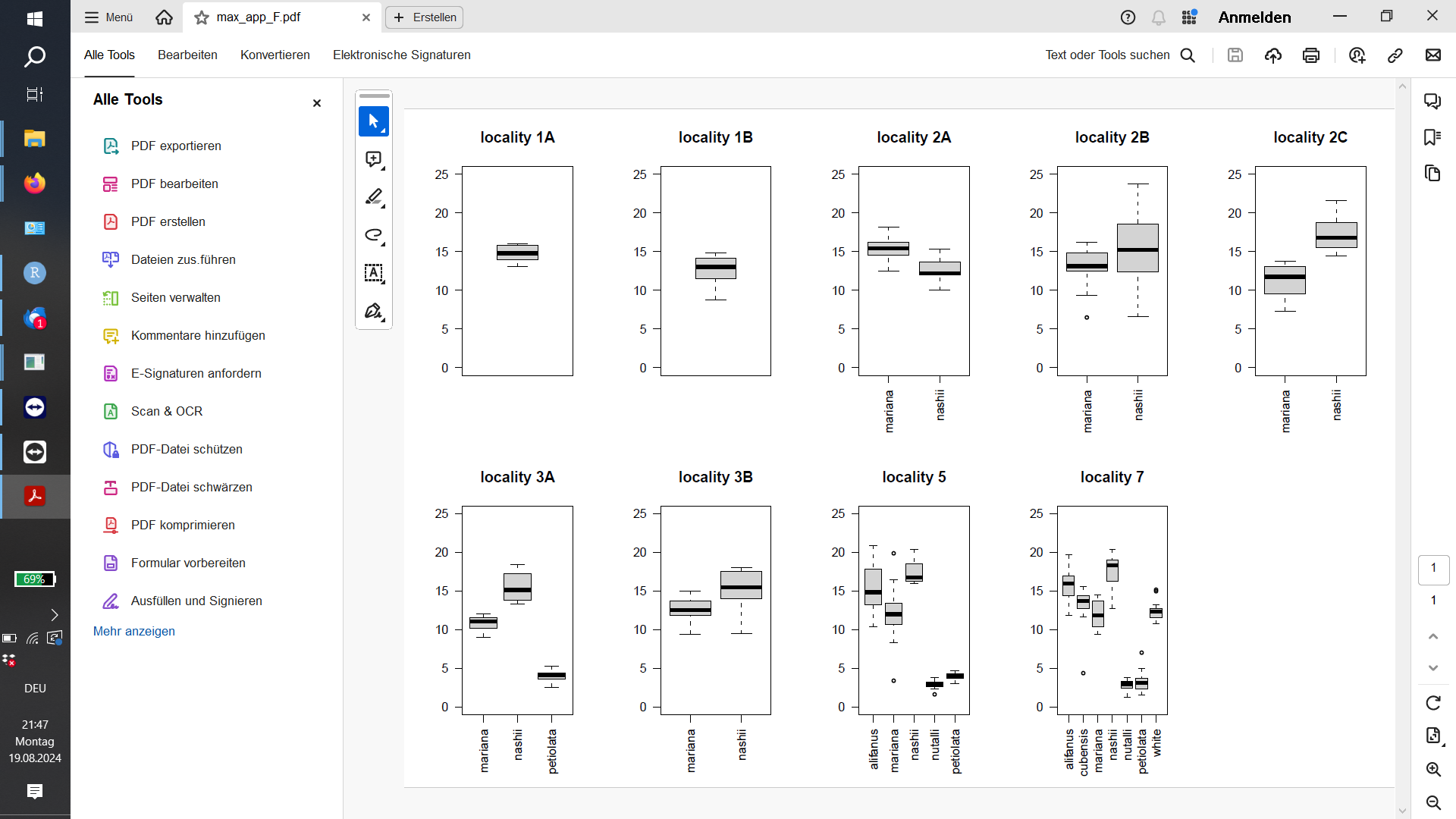


**Figure S6. Maximum anther-base stigma distance for the study species across localities**, with *R. mariana* taking up an intermediate position.


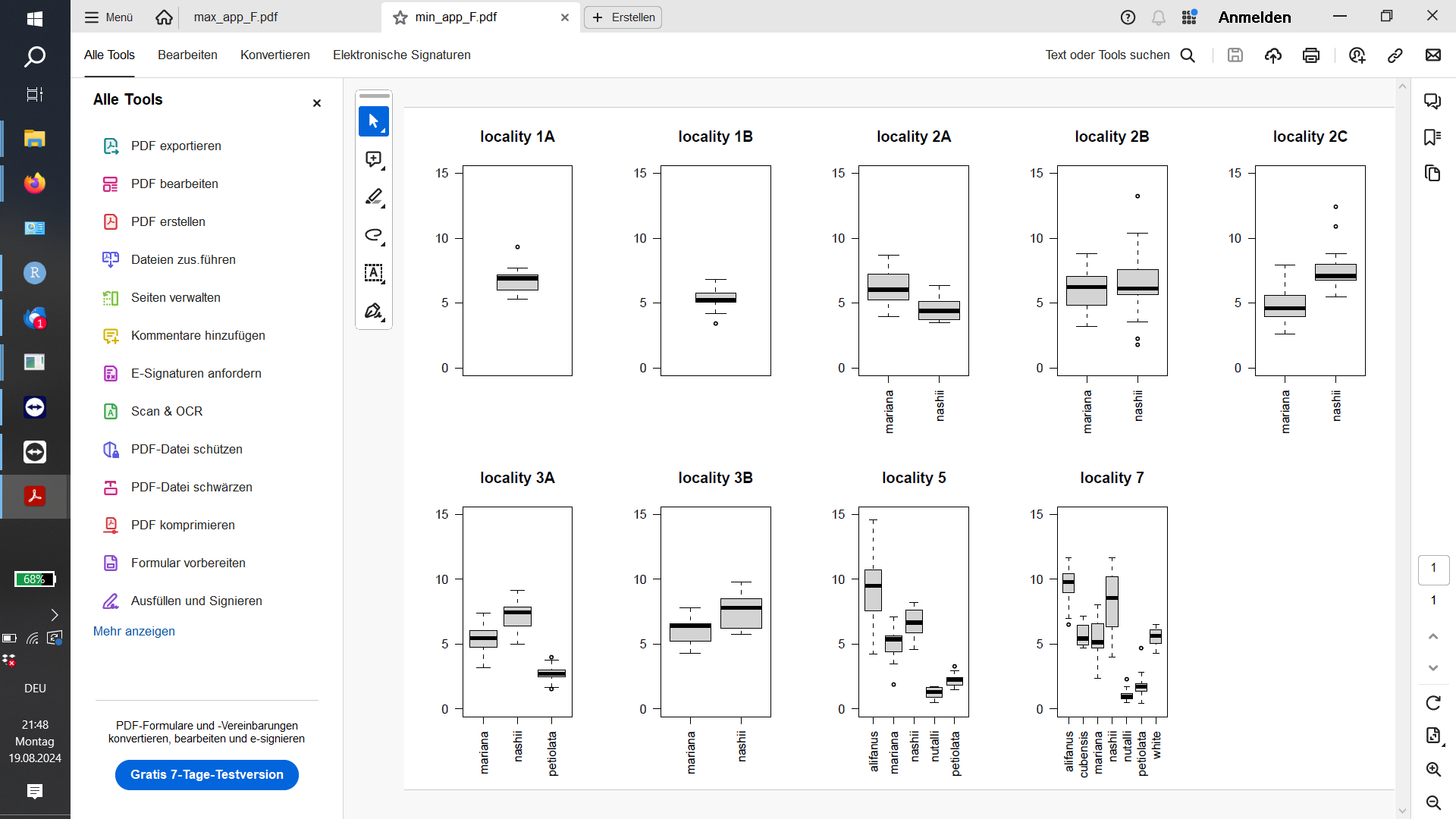


**Figure S7. Minimum anther-base stigma distance for the study species across all localities**, with *R. mariana* taking up an intermediate position.

**Table S11. Kruskal-Wallis ANOVA on maximum and minimum distance between stamen base and stigma** among co-flowering species at each locality. Significant differences in this trait indicate the potential for differential size-matching with different buzzing bees.

|  | **maximum distance** | | **minimum distance** | |
| --- | --- | --- | --- | --- |
| **locality** | **Chi²** | **p-value** | **Chi²** | **p-value** |
| **2A** | 13.93 | <0.001 | 11.15 | < 0.001 |
| **2B** | 3.30 | 0.06896 | 0.30 | 0.58 |
| **2C** | 19.28 | <0.001 | 12.0 | <0.001 |
| **3A** | 39.13 | <0.001 | 34.2 | <0.001 |
| **3B** | 11.71 | <0.001 | 8.31 | 0.004 |
| **5** | 52.41 | <0.001 | 55.34 | <0.001 |
| **7** | 72.67 | <0.001 | 72.42 | <0.001 |

**Table S12. Dunn-test results on pairwise differences among species in multi-species sites 3A, 5 and 7,** p-values smaller than 0.01 are indicated by an *, non-significant p-values are indicated by ‘ns’.

| **maximum distance** | |  |  |  |  |  |
| --- | --- | --- | --- | --- | --- | --- |
| **3A** | **mariana** | nashii |  |  |  |  |
| nashii | ***** |  |  |  |  |  |
| petiolata | ***** | * |  |  |  |  |
| **5** | alifanus | **mariana** | nashii | nuttallii |  |  |
| **mariana** | **ns** |  |  |  |  |  |
| nashii | ns | ***** |  |  |  |  |
| nuttallii | * | ***** | * |  |  |  |
| petiolata | * | ***** | * | ns |  |  |
| **7** | alifanus | cubensis | **mariana** | nashii | nuttallii | petiolata |
| cubensis | ns |  |  |  |  |  |
| **mariana** | **0.03** | **ns** |  |  |  |  |
| nashii | ns | 0.02 | **0.01** |  |  |  |
| nuttallii | * | * | **0.01** | * |  |  |
| petiolata | * | * | **0.02** | * | ns |  |
| var. exalbida | * | ns | **ns** | * | * | * |
| **minimum distance** | |  |  |  |  |  |
| **3A** | **mariana** | nashii |  |  |  |  |
| nashii | ***** |  |  |  |  |  |
| petiolata | ***** | * |  |  |  |  |
| **5** | alifanus | **mariana** | nashii | nuttallii |  |  |
| **mariana** | ***** |  |  |  |  |  |
| nashii | ns | **0.03** |  |  |  |  |
| nuttallii | * | ***** | * |  |  |  |
| petiolata | * | ***** | * | ns |  |  |
| **7** | alifanus | cubensis | **mariana** | nashii | nuttallii | petiolata |
| cubensis | * |  |  |  |  |  |
| **mariana** | **0.02** | **ns** |  |  |  |  |
| nashii | ns | 0.04 | **ns** |  |  |  |
| nuttallii | * | * | ***** | * |  |  |
| petiolata | * | * | **0.02** | * | ns |  |
| var. exalbida | * | ns | **ns** | 0.02 | * | * |


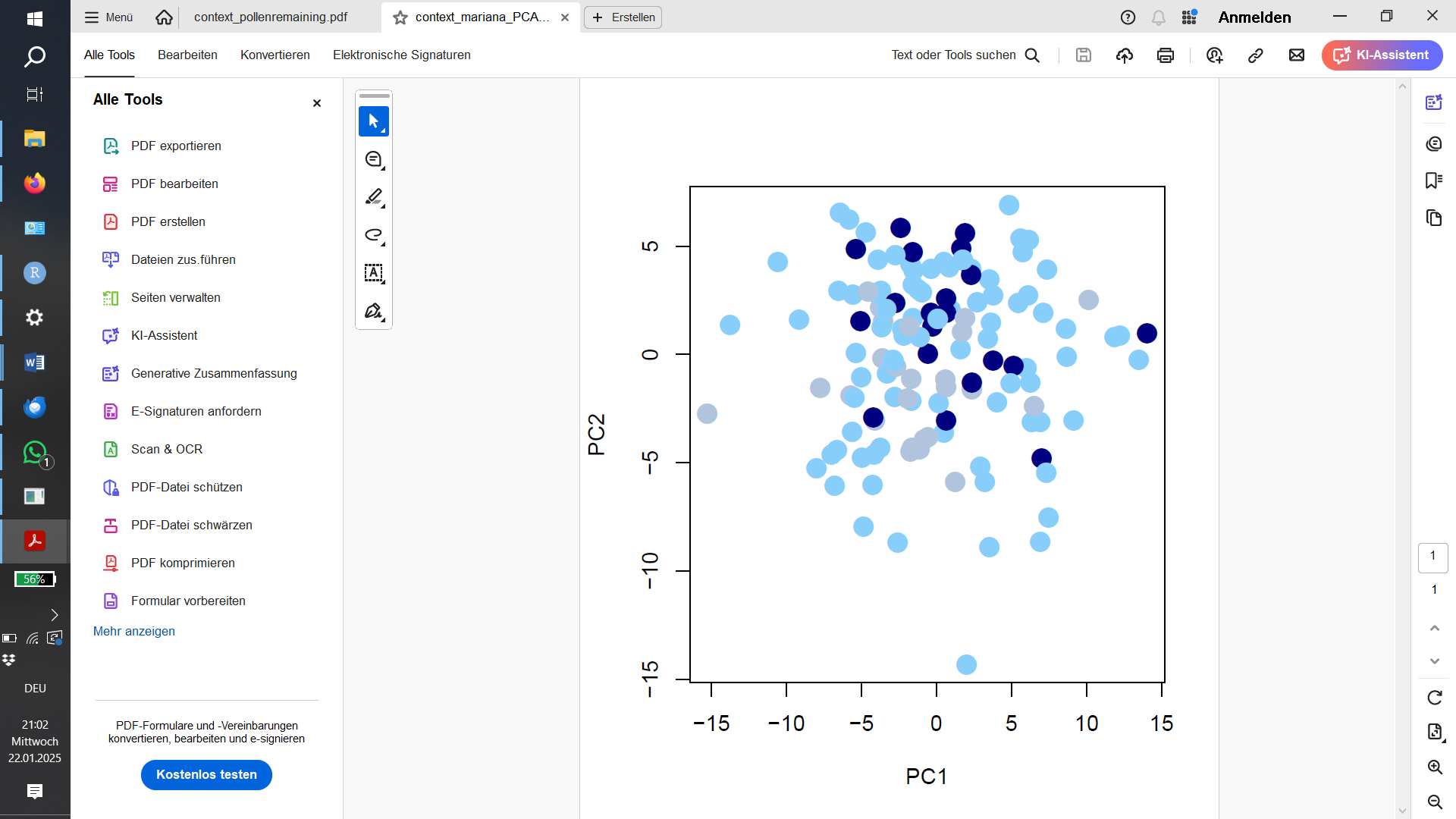


**Figure S8. Floral trait space of *R. mariana*** comparing low (grey, single-species localities), intermediate (light blue, 2-3 species co-flowering) and high (dark blue, 5-7 species co-flowering) co-flowering contexts. Low and high co-flowering contexts were significantly different from each other.

**Table S13. Pairwise differences in floral trait space occupation among localities of *R. mariana***. Significant differences are highlighted in bold.

| **pairs** | |  | **Sums of sqs** | **F.Model** | **R2** | **p.adjusted** |
| --- | --- | --- | --- | --- | --- | --- |
| 1A | vs | 2B | 316.06462 | 6.5010538 | 0.22036683 | 0.108 |
| **1A** | **vs** | **3A** | **465.46193** | **11.4541676** | **0.23639179** | **0.036** |
| 1A | vs | 7 | 151.60963 | 6.3165932 | 0.32700348 | 0.072 |
| **1A** | **vs** | **5** | **284.47206** | **6.7805460** | **0.22768374** | **0.036** |
| 1A | vs | 2C | 106.76657 | 2.8747279 | 0.11110176 | 1.000 |
| 1A | vs | 2A | 110.27129 | 2.6183728 | 0.10220683 | 1.000 |
| **1A** | **vs** | **1B** | **156.40690** | **5.9769031** | **0.20626438** | **0.036** |
| **2B** | **vs** | **3A** | **494.29434** | **9.2080811** | **0.17981695** | **0.036** |
| 2B | vs | 7 | 53.50304 | 0.9059452 | 0.04791854 | 1.000 |
| **2B** | **vs** | **5** | **546.93971** | **8.9246283** | **0.24169853** | **0.036** |
| **2B** | **vs** | **3A** | **724.21544** | **12.6324887** | **0.31089626** | **0.036** |
| **2B** | **vs** | **2A** | **215.17342** | **3.5035416** | **0.11121104** | **0.396** |
| **2B** | **vs** | **1B** | **610.39282** | **12.6329372** | **0.31090386** | **0.036** |
| 3A | vs | 7 | 110.34297 | 2.4378402 | 0.07078958 | 1.000 |
| 3A | vs | 5 | 172.16452 | 3.4411192 | 0.07572699 | 0.612 |
| **3A** | **vs** | **2C** | **557.56649** | **11.7642300** | **0.21881147** | **0.036** |
| **3A** | **vs** | **2A** | **339.15948** | **6.7670404** | **0.13876258** | **0.036** |
| **3A** | **vs** | **1B** | **1064.01405** | **25.7088625** | **0.37969716** | **0.036** |
| 7 | vs | 5 | 170.81668 | 3.3795974 | 0.15807582 | 0.504 |
| 7 | vs | 2C | 240.41065 | 5.4156649 | 0.23128384 | 0.468 |
| 7 | vs | 2A | 83.70291 | 1.6493736 | 0.08394026 | 1.000 |
| **7** | **vs** | **1B** | **332.99057** | **10.9633714** | **0.37852539** | **0.036** |
| 2C | vs | 5 | 175.75131 | 3.2707206 | 0.11569286 | 0.144 |
| 5 | vs | 2C | 225.57740 | 4.3500585 | 0.13446834 | 0.072 |
| 5 | vs | 2A | 178.07584 | 3.1831911 | 0.10208035 | 0.360 |
| **5** | **vs** | **1B** | **471.30167** | **11.0003736** | **0.28205816** | **0.036** |
| 2C | vs | 2A | 237.56404 | 4.5696082 | 0.14030283 | 0.252 |
| **2C** | **vs** | **1B** | **368.20178** | **9.4679135** | **0.25269391** | **0.036** |
| 2A | vs | 1B | 226.45298 | 5.2693124 | 0.15838357 | 0.108 |


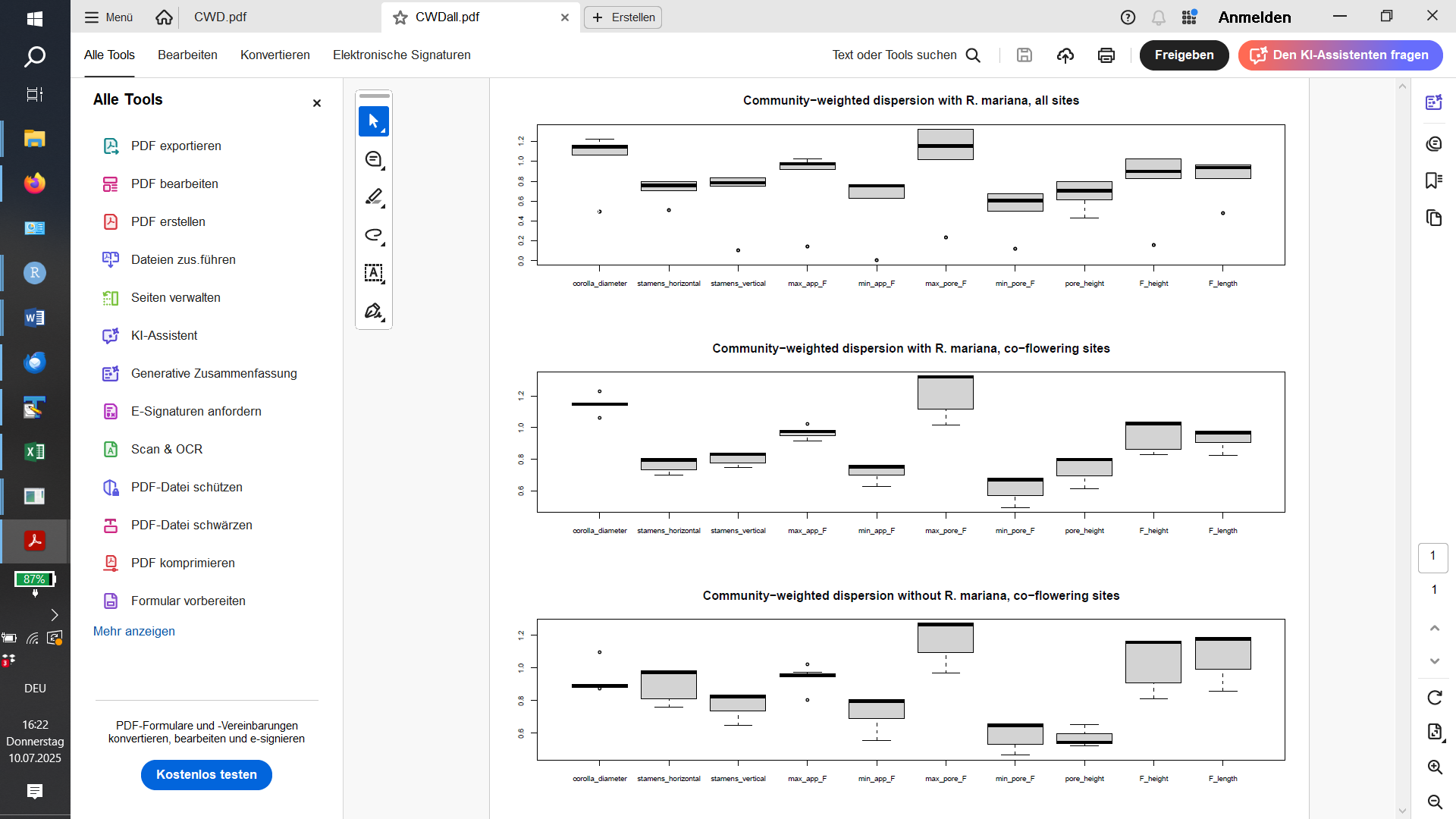


**Figure S9. Community-weighted dispersion of floral traits** calculated for all sites and co-flowering sites, the latter with and without *R. mariana*; removing *R. mariana* from the dataset allows to evaluate its contribution to community-wide dispersion for reach trait. Note that the low dispersion (outliers) in the upper graph stem from the single-flowering localities of *R. mariana*.
